# The presence of multiple variants of IncF plasmid alleles in a single genome sequence can hinder accurate replicon sequence typing using *in silico* pMLST tools

**DOI:** 10.1128/msystems.01010-24

**Published:** 2025-04-08

**Authors:** Michaela Ruzickova, Jana Palkovicova, Ivo Papousek, Max L. Cummins, Steven P. Djordjevic, Monika Dolejska

**Affiliations:** 1 Central European Institute of Technology, University of Veterinary Sciences Brno 48358, Brno, South Moravian Region, Czechia; 2 Department of Biology and Wildlife Diseases, Faculty of Veterinary Hygiene and Ecology, University of Veterinary Sciences Brno 183749, Brno, South Moravian Region, Czechia; 3 Department of Microbiology, Faculty of Medicine in Pilsen, Charles University, Pilsen, Czechia; 4 Australian Institute for Microbiology and Infection, University of Technology Sydney 1994, Sydney, New South Wales, Australia; 5 The Australian Centre for Genomic Epidemiological Microbiology, University of Technology Sydney 1994, Sydney, New South Wales, Australia; 6 Division of Clinical Microbiology and Immunology, Department of Laboratory Medicine, The University Hospital Brno 48243 , Brno, South Moravian Region, Czechia; 7 Department of Chemistry and Biochemistry, Faculty of AgriSciences, Mendel University in Brno 309613, Brno, South Moravian Region, Czechia; Institute of Biochemistry and Biophysics of the Polish Academy of Sciences, Warsaw, Poland

**Keywords:** plasmids, IncF, pMLST, Enterobacteriaceae, antibiotic resistance

## Abstract

IncF plasmids are mobile genetic elements found in bacteria from the *Enterobacteriaceae* family and often carry critical antibiotic and virulence gene cargo. The classification of IncF plasmids using the plasmid Multi-Locus Sequence Typing (pMLST) tool from the Center for Genomic Epidemiology (CGE; https://www.genomicepidemiology.org/) compares the sequences of IncF alleles against a database to create a plasmid sequence type (ST). Accurate identification of plasmid STs is useful as it enables an assessment of IncF plasmid lineages associated with pandemic enterobacterial STs. Our initial observations showed discrepancies in IncF allele variants reported by pMLST in a collection of 898 *Escherichia coli* ST131 genomes. To evaluate the limitations of the pMLST tool, we interrogated an in-house and public repository of 70,324 *E. coli* genomes of various STs and other *Enterobacteriaceae* genomes (*n* = 1247). All short-read assemblies and representatives selected for long-read sequencing were used to assess pMLST allele variants and to compare the output of pMLST tool versions. When multiple allele variants occurred in a single bacterial genome, the Python and web versions of the tool randomly selected one allele to report, leading to limited and inaccurate ST identification. Discrepancies were detected in 5,804 of 72,469 genomes (8.01%). Long-read sequencing of 27 genomes confirmed multiple IncF allele variants on one plasmid or two separate IncF plasmids in a single bacterial cell. The pMLST tool was unable to accurately distinguish allele variants and their location on replicons using short-read genome assemblies, or long-read genome assemblies if the same allele variant was present more than once.

**IMPORTANCE:**

Plasmid sequence type is crucial for describing IncF plasmids due to their capacity to carry important antibiotic and virulence gene cargo and consequently due to their association with disease-causing enterobacterial lineages exhibiting resistance to clinically relevant antibiotics in humans and food-producing animals. As a result, precise reporting of IncF allele variants in IncF plasmids is necessary. Comparison of the FAB formulae generated by the pMLST tool with annotated long-read genome assemblies identified inconsistencies, including examples where multiple IncF allele variants were present on the same plasmid but missing in the FAB formula, or in cases where two IncF plasmids were detected in one bacterial cell, and the pMLST output provided information only about one plasmid. Such inconsistencies may cloud interpretation of IncF plasmid replicon type in specific bacterial lineages or inaccurate assumptions of host strain clonality.

## INTRODUCTION

Plasmids of incompatibility group F (IncF) are diverse mobile genetic elements widely found in various bacteria from the *Enterobacteriaceae* family (1). Due to their conjugative abilities, they are horizontally transferred among such species, which increases their potential impact on bacterial evolution (2). IncF plasmids are multi-replicon plasmids that usually carry more than one replicon responsible for the initiation of plasmid DNA replication. The mosaic structure of the IncF plasmids may have implications for broader host range replication (3, 4). Replicons located on typical IncF plasmids belong to FII, FIA, and FIB families with the latter two being the ones predominantly used for initiating replication, which leaves the FII replicon free to be used when incompatibility obstacles arise (3). If a cell contains two plasmids, both carrying FII, FIA, and FIB replicons, each plasmid can use a different replicon for its replication allowing both of them to be stably maintained in the cell (3, 5).

The CGE pMLST *in silico* tool is often used to further classify plasmids. It targets specific sequences linked to the regions of replication proteins in plasmid genomes and uses BLASTn to compare them against a database of known replicon alleles (6). To classify IncF plasmids, a scheme called replicon sequence typing (RST) was established within the pMLST tool based on the variety of known IncF replicon alleles. The output of this analysis, known as the FAB formula, is created by the allele variants for each of the plasmid replicons FII, FIA, and FIB, respectively, and can be used for plasmid identification. However, variations of these three basic replicons also exist within the formula, such as in the FII replicon, which belongs to a larger FII family together with FII_K_, FII,_S_ and FII_Y_ alleles being mostly species-specific for *Klebsiella* sp., *Salmonella* sp., and *Yersinia* sp., respectively. Its place in the formula can be also taken by the FIC replicon from the same FII family, which occurs regardless of the species (5, 7).

Precise calling of replicons and allele variants present in mosaic IncF plasmids is of high importance not only for deciphering their evolution but also for the epidemiology of the clinically relevant plasmid types responsible for dissemination of antibiotic resistance and virulence genes (8). The FAB formula is a significant parameter for determining the association of IncF plasmids with certain bacterial species and genotypes, and omission of some of the alleles may lead to improper evaluation of clonality among bacterial strains (5, 9).

During the analysis of IncF plasmid content in a large collection of *E. coli* ST131 isolates, inconsistent results of repeated RST analysis were observed in regard to allele variants of plasmid replicons that define the FAB formula. The ST131 lineage was selected for the study due to a large number of available genome sequences with sublineages often associated with specific IncF RST and due to its clinical importance. The main aims were to (i) assess inconsistencies in IncF RST, (ii) identify plasmid lineages impacted by each discrepancy, (iii) identify specific alleles by analyzing an extensive collection of *E. coli* genomes (*n* = 70,324) as well as other species from the *Enterobacteriaceae* family (*n* = 1,247), and (iv) examine complete plasmid sequences from our in-house collection as well as publicly available databases (*n* = 7,441) for inconsistency in the FAB formulae.

## MATERIALS AND METHODS

### Collection of bacterial genomes

Our initial observations identified discrepancies in the identified replicon alleles in our in-house collection of Czech *E. coli* ST131 genomes (*n* = 898) from human (79.40%; 713/898), animal (5.12%; 46/898), and environmental sources (15.48%; 139/898) (published under BioProject PRJNA983524 and PRJNA595483 [10]). Since a detailed pMLST analysis pointed out inaccurate reporting of the IncF allele variants, more sequences were assessed to evaluate aberrant pMLST calling within a larger cohort of ST131 genome sequences and more broadly among *E. coli*. This was done to exclude the possibility of a technical error and avoid potential bias from our collection originating from a single country and a single *E. coli* ST. These analyses included the international collection of *E. coli* ST131 genomes (*n* = 13,656), which was accessed from Enterobase (https://enterobase.warwick.ac.uk/) on 21 February 2023. Moreover, 121,427 genomes of various *E. coli* STs were also obtained from Enterobase on 30 May 2023 together with their metadata to assess the frequency of inconsistent IncF RST replicon types in diverse *E. coli* STs. Sequences with missing metadata (source, country, year) and those which did not pass quality checks were excluded from our analyses. *E. coli* ST131 genomes were also excluded from the broader *E. coli* collection as they were already assessed separately. Quality checks considered the number of contigs (threshold 400 contigs) and the total length of the genome (threshold 4,500,000 base pairs). In addition, non-ST131 *E. coli* isolates (*n* = 1,743) from our in-house collection were also included. The entire collection sourced from Enterobase comprised 8,514 *E. coli* ST131 genomes and 60,067 sequences representative of diverse *E. coli* STs.

Finally, to check for this phenomenon in genera other than *E. coli*, an in-house collection of whole-genome sequences of *Enterobacteriaceae* (*n* = 2,342) accumulated during previous projects was used for the assessment of aberrant IncF plasmid replicon calls. Genomes with unreliable species affiliation or that failed to meet our quality criteria as specified above were excluded. The final collection consisted of 1,247 genomes.

Raw data from short-read sequencing of each in-house collection were quality (Q ≥ 20) and adaptor trimmed, and *de novo* assembled with minimum contig length set to 200 base pairs. This was followed by the quality and contamination check. Downloaded publicly available short-read assemblies went through quality and contamination checks. The presence of plasmids in these genomes was determined using ABRicate (https://github.com/tseemann/abricate) and the PlasmidFinder database with coverage and identity threshold set to 90% for the presence of plasmid replicons (6). Non-specified parameters were set to default. All the kits, platforms, and software used for short-read sequencing, assembly, quality and contamination check, and data processing are described in Table S1.

### Collection of complete plasmid sequences

Complete IncF plasmids (*n* = 7,441) were accessed on 27 January 2023. Reference sequences of FII, FIA, FIB, FIC, FII_K_, FII,_S_ and FII_Y_ alleles downloaded from PubMLST (https://pubmlst.org/) were used as query sequences to search for the corresponding plasmid genomes in the NCBI database (11). No filtering based on metadata was done in this collection since we did not intend to use the data for epidemiological comparison. These sequences obtained from a public database were not run through quality check as they were considered complete plasmid sequences. Data from all of the collections can be seen in Table S1 together with all the kits, platforms, and software used for long-read sequencing and data processing.

To test hypotheses based on short-read genome assemblies’ analyses, accurately determine plasmid replicons, and obtain complete plasmid sequences, several representative *E. coli* ST131 isolates from the in-house collection (*n* = 27) were also subjected to long-read sequencing. The representatives were selected based on the multiple allele variants detected in the short-read genome assemblies, presence of antibiotic resistance genes, and their clonality. When selecting strains with the same IncF RST profile, isolates originating from varying sources and years were prioritized. Long-read sequencing was necessary to confirm the presence of alleles identified by pMLST and identify the location of multiple allele variants in the genome. Genomic DNA of the selected isolates was extracted and used for the preparation of sequence libraries according to the manufacturer’s protocol. The mixture was subsequently loaded onto a flow cell and sequenced on a MinION platform (Oxford Nanopore Technologies, UK). The long-read raw data obtained by base-calling under high-accuracy model were adaptor-trimmed, demultiplexed, and quality-trimmed (Q ≥ 9). The long reads were *de novo* assembled, and the obtained assemblies were polished using both corrected long reads and corrected short reads. Parameters of all the tools were set to default unless specified otherwise. Obtained genomes were used for the detection of IncF allele variants. The plasmids then underwent automatic annotation with manual supervision. Annotated long-read assemblies are available on GenBank under accession numbers PQ066878–PQ066914.

### pMLST tool versions used for the analyses

All genome and plasmid sequences were subjected to IncF plasmid typing by pMLST analysis using three different versions of the pMLST tool. Web-based tool pMLST 2.0 from CGE (https://www.genomicepidemiology.org/) as well as two versions of downloadable python pMLST software (https://bitbucket.org/genomicepidemiology/pmlst/) pulled either from Docker (https://github.com/docker) or Anaconda (https://github.com/conda) repositories were used for the RST of IncF plasmids. The web-based tool provides a table of hits for each allele, including identity and coverage values, and creates the FAB formula from the hits. The output of both versions is text files for each of the analyzed isolates that show the allele hits together with their identity and coverage and suggest the FAB formula. The FAB formula is then reported as a “Sequence type” of the plasmid by all of the pMLST tools.

## RESULTS AND DISCUSSION

### Current pMLST tools fail to consistently assign multiple IncF replicons

Inconsistent results regarding the FAB formula were observed during repeated pMLST analysis using three different versions of the CGE pMLST tool. All of them are capable of finding two of the allele variants if they are both present in either one of the FII, FIA, and FIB replicons. The tool flags instances where more than one allele occurs in a single replicon, with a warning “FII/FIA/FIB: Multiple perfect hits found.” The tool, irrespective of which version is selected, generates a single FAB formula created by randomly selecting one of the multiple possible hits. Consequently, the output does not accurately reflect the allele content found by the tool and fails to accurately predict the specific plasmid(s). Instances of genomes carrying three different allele variants or two copies of the same allele variant on a single FII, FIA, or FIB replicon were also observed; however, these were not confirmed by long-read sequencing. These situations were both detected only by the web-based version of the tool. Surprisingly, the Python versions did not display the extra alleles as they were able to recognize only one alternative hit. The information about the multiple FII allele hits is stored in the detailed results of the pMLST analysis in all three versions of the tool, see Data S1 to S3. This information can be extracted and added manually to an FAB formula but is not automatically included in the final plasmid ST. Even though we extract it manually, this information does not necessarily enable an accurate identification of the IncF plasmid replicon(s) present in the bacterial genomes. Of the collection of 72,469 genomes, 5,804 (8.01%) displayed discrepancies in the pMLST output.

Other tools used for plasmid identification are PubMLST (https://pubmlst.org/) and Plasmid Taxonomic Unit (PTU [12]). Since the PTU focuses more on searching the genome for the plasmid itself, it is not routinely used for the plasmid ST classification and thus was not suitable for our study. PubMLST was used for running the representative isolates mentioned below. This tool did identify the same alleles as the CGE pMLST tool, but it was also unable to distinguish the presence of multiple plasmids. The PubMLST tool also does not create the FAB formula.

### Two FII alleles on a single plasmid

**Fig 1:**
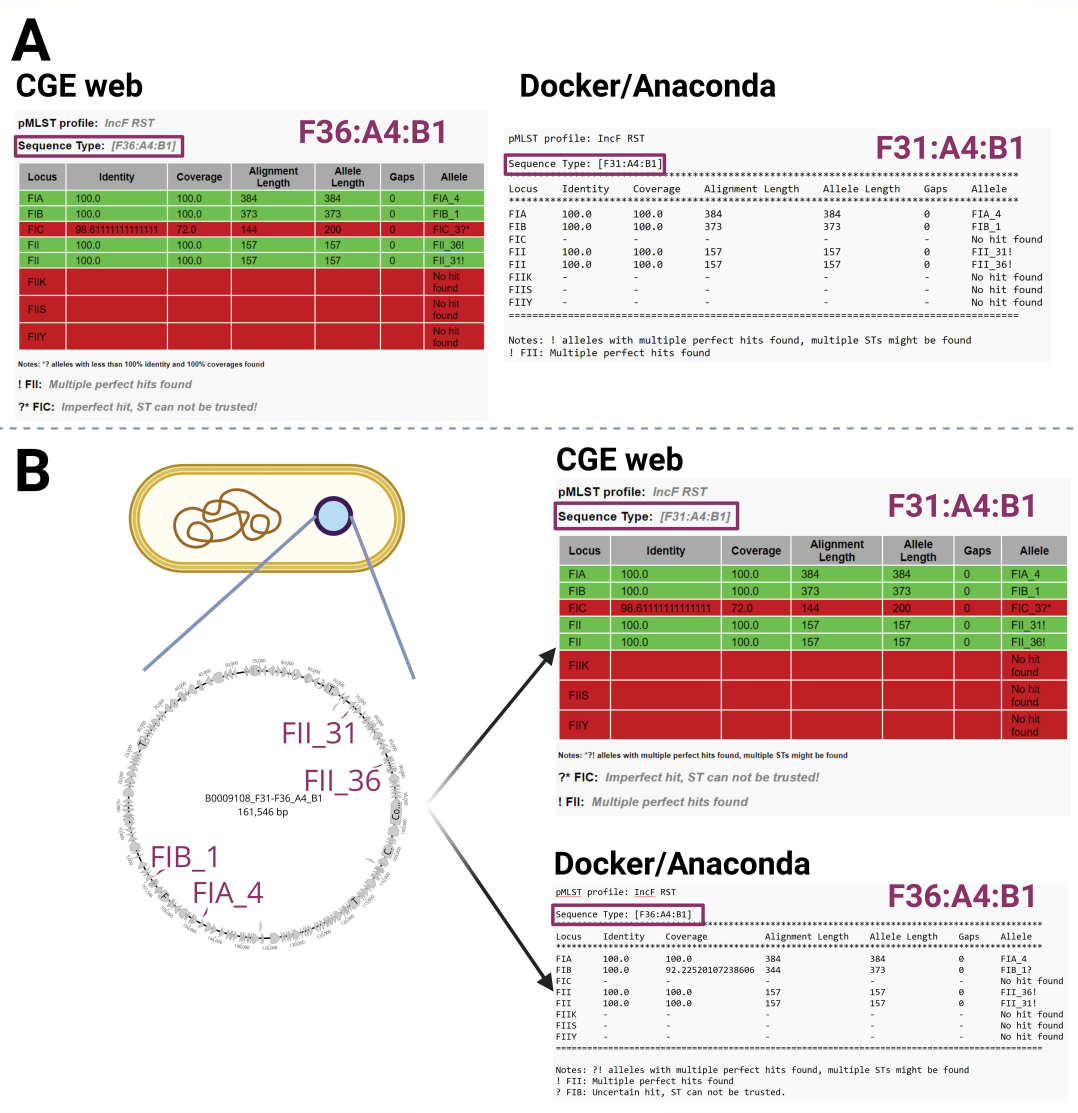
Presence of two FII alleles located on a single plasmid. Inconsistency between pMLST output of short-read assemblies analysis using three versions of the tool with one of the FII allele variants being omitted while creating the FAB formula (A) and the results of long-read assembly analysis showing presence of an F31/F36:A4:B1 plasmid carrying both F31 and F36 alleles with an incorrectly assigned plasmid ST (B).

Several situations may account for the discrepancies observed when multiple allele hits were detected. In one scenario, all of the alleles are present on one plasmid, thus the information about the epidemiological background of a plasmid is incomplete. The FAB formula created by the pMLST analysis of short-read genome assemblies displays two different outputs varying between F31:A4:B1 and F36:A4:B1 as highlighted in Fig. 1. The detailed output may predict the presence of both of these alleles in all the versions of the tool together with a warning that multiple perfect hits were found (Fig. 1A). Regarding long-read analysis, this situation also occurred for the allele variant combination F31/F36, either in F31/F36:A4:B1, F31/F36:A-:B1, or F31/F36:A4:B58 plasmids. All the representative isolates carrying the FII alleles F31 and/or F36 that underwent long-read sequencing (*n* = 18) confirmed carriage of both of these alleles on the same plasmid. The allele combination F31/F36 was observed in 6.20% (360/5804) of all short-read sequenced isolates with multiple allele variants; however, the frequency of carriage of these alleles on a single plasmid would need to be confirmed by long-read sequencing. The formula F31/F36:A4:B1 can be seen in isolate B0009108 with both of the FII allele variants being carried on a single plasmid (Fig. 1B). The FAB formula suggested by the pMLST tool predicts F31:A4:B1 and F36:A4:B1 when the accurate output should state F31/F36:A4:B1.

### Two IncF plasmids present in one cell reported as a single plasmid

**Fig 2:**
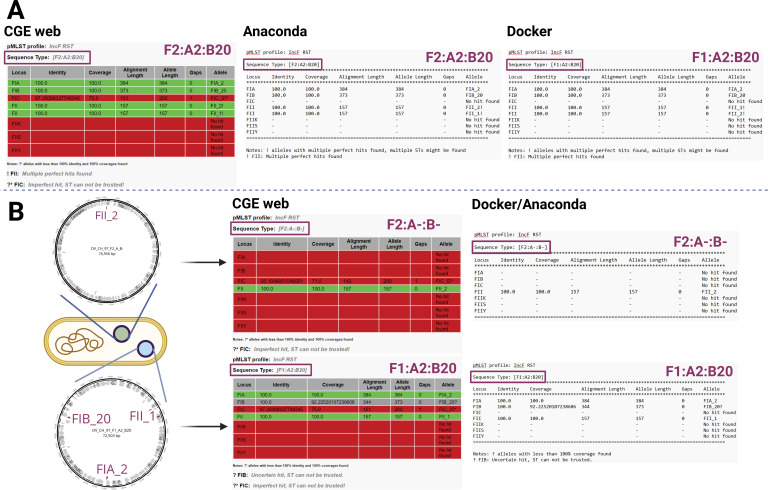
Presence of two IncF plasmids located in one cell with an incorrect FAB formula identification. The discrepancies observed during the analysis of the short-read genome assemblies using three versions of pMLST, which resulted in different plasmid STs for the same isolate (A), and the results obtained from long-read sequencing showing two plasmids, F1:A2:B20 and F2:A-:B-, being carried by the cell and a correct assignment of FAB formula (B). The FIC allele shown in the output was not taken into consideration due to its identity being lower than 100.00% and its coverage reaching only to 75.00%.

A second scenario where two or more alleles are located on different plasmids (Fig. 2) was also observed. None of the analyses of short-read genome assemblies pointed out the possibility of multiple plasmids occurring in the cell. This situation highlights how associations between plasmids and antibiotic resistance genes could be inaccurate, since the true ST of the plasmid may not reflect the ST predicted by the *in silico* pMLST tool, as was demonstrated in the short-read assembly of isolate OV_CH_97. The pMLST output predicted F1:A2:B20 and F2:A2:B20 (Fig. 2A), whereas the genomic analysis of long-read genome assemblies of this isolate showed a presence of two IncF plasmids (Fig. 2B). After obtaining two different plasmids, pMLST was run on both of their sequences separately with two FAB formulae describing the respective plasmids as F1:A2:B20 and F2:A-:B-, with both of them carrying the complete IncF plasmid backbone. When the pMLST was run on the complete long-read genome assembly only, the output did not differ from the one obtained by analysing short-read assemblies, with both inaccurately predicting plasmid sequence type. In summary, the RST formula F1/F2:A2:B20 predicted from short-read data reflects the allele variants present in the cell but not the fact that two IncF plasmids reside in the cell. A similar situation was observed in isolate U23060 (Table S1). Both tools predicted the FAB formula F29:A-:B10 with both short and long-read analyses. Isolate U23060 has shown to contain two plasmids with F29:A-:B10 and K7:A-:B- formulae with the FIIK allele identified only when running pMLST on the two plasmid sequences separately. These data confirm that the pMLST tools are sometimes unable to identify the full plasmid cohort using both short-read and long-read-generated genome assemblies.

### Overlapping FII and FIC alleles on the same plasmid adversely impact RST assignment

**Fig 3:**
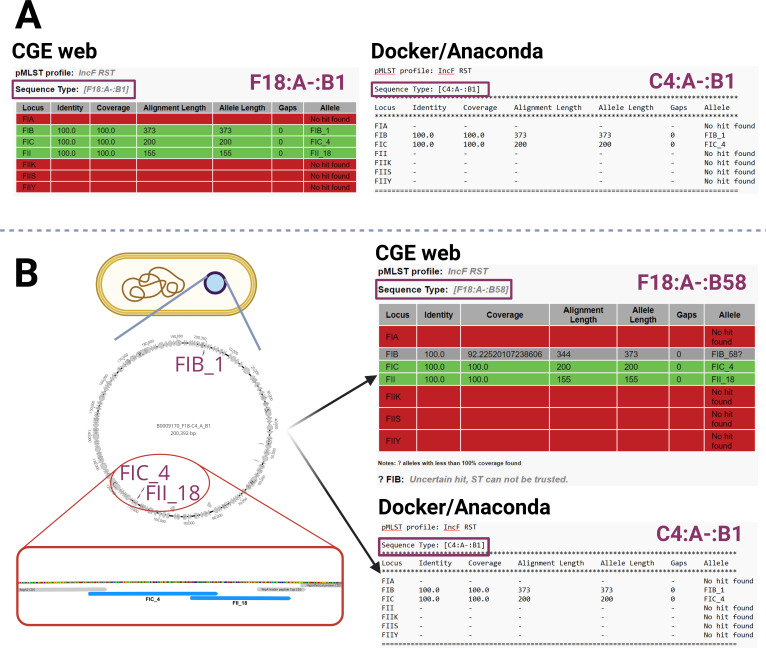
Presence of an FII and FIC allele overlapping one another on a single plasmid. Conflicting results were obtained from short-read assemblies using CGE web pMLST versions, which showed the presence of the F18 allele, while the Docker/Anaconda versions omitted it completely (A). Long-read assembly analysis displayed the same discrepancy between versions and showed a complete plasmid carrying both C4 and F18 alleles with an overlap (B).

In a third scenario, the start of the FII replicon overlaps with the end of the sequence of the FIC replicon in the same orientation, with both of them being located on a single plasmid (Fig. 3). Even though both of these alleles, FIC and FII, are present in the cell with 100% identity and 100% coverage, only one of them can take the position of the FII allele in the FAB formula with the selection being random. The presence of both alleles was detected only by the CGE version of the pMLST tool, while the Docker and Anaconda versions omitted the FII allele. Short-read assembly of isolate B0009170 provided two pMLST outputs—FAB formulae C4:A-:B1 and F18:A-:B1 (Fig. 3A). Analysis of the long-read genome assemblies showed both F18 and C4 allele variants present on a single C4/F18:A-:B1 plasmid with the alleles overlapping in one region (Fig. 3B). In this case, the C4 (200 bp sequence) and F18 (155 bp sequence) allele variants display an overlap of 43 bp. Moreover, the allele variant F18 was detected only by the CGE web version in the analysis of both short-read and long-read assemblies with Docker and Anaconda versions omitting this data. The situation with the C4/F18 allele combination occurred in 5.48% (318/5804) of all short-read genome assemblies carrying multiple allele variants. Short-read genome assemblies belonging to *E. coli* ST131 (*n* = 18) and carrying the C4/F18 combination were mapped to the plasmid sequence obtained by long-read sequencing using Geneious mapper under Geneious Prime 2024.0.3 (https://www.geneious.com). The overlap of C4 and F18 alleles was confirmed in all 18 assemblies connecting this combination of alleles to the overlapping scenario. The discrepancy between B1 and B58 allele variants seen in Fig. 3B is addressed later as the phenomenon occurs across various plasmids.

### The difference between two FIB alleles is obfuscated due to the circular plasmid genome being severed at the replication origin

**Fig 4:**
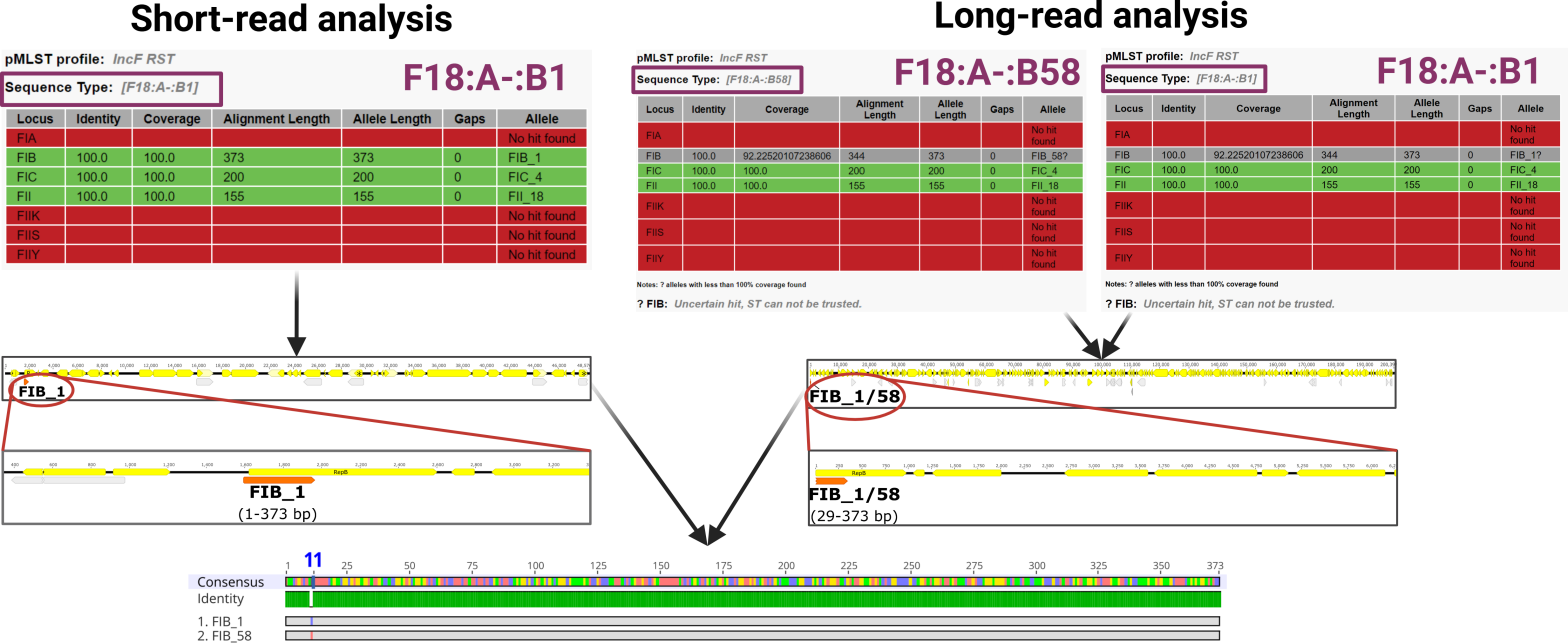
Discrepancies in the FIB allele variant observed between short-read and long-read analyses. Short-read analysis shows the presence of the B1 allele with 100% coverage and 100% identity due to the complete allele being present in the middle of a contig. Repeated pMLST analysis of long-read assembly provided two different alleles, B1 and B58, which proved to differ in a single nucleotide located 11 nucleotides from the start of the allele sequence. Due to the circular nature of the complete plasmid, the sequence begins from the start of the replication protein, causing the omission of the 29 bp region at the start of the allele, which contains the varying 11th nucleotide of the allele sequence. All three pMLST tool versions provided the same results; therefore, only the CGE web output is shown.

Finally, discrepancies were found in the FIB allele variant of multiple RSTs (Fig. 4). This was observed in the isolate B0009170 (see Fig. 3) where the FAB formula showed either the allele B1 or B58. In short-read genome assemblies, all pMLST tools correctly reported a B1 allele with 100% identity and 100% coverage, but the pMLST analysis of complete plasmids resulted in randomly assigned B1 or B58 with 100% identity and 92.23% coverage. The alignment of these two FIB allele variants showed identical length and a single nucleotide difference located on the 11th nucleotide of their genome. In plasmids, the sequence of either one of these alleles starts 29 bp upstream of the replication protein. As there are only contigs and not circular genomes in *de novo* assemblies from short-read sequencing, the replication proteins along with the IncF allele sequences are usually in the middle of a contig. However, the long-read plasmid assemblies are typically complete circular genomes and thus are rotated during assembly to have the replication protein, i.e., the replication origin, at the beginning of a plasmid (13). Since the pMLST tools work only with linear sequences, the circular plasmid sequence is severed at the replication origin at the start of the replication protein, effectively omitting the first 29 bp at the beginning of the allele from the RST assessment, which causes an incorrect plasmid identification (Fig. 4).

These observations stress the importance of assessing the detailed outputs generated by pMLST tools as the automatically generated FAB formula occasionally omits calling allele variants in IncF plasmid sequences and miscalls the pMLST. These omissions obfuscate the ability to accurately interpret plasmid epidemiology and phylogeny and their association with bacterial clones and antimicrobial resistance genes. When analyzing the pMLST output, we recommend not to rely on the RST output but to consider the allele variants generated by the tool. Unfortunately, long-read sequencing is needed in order to detect all of the allele variants in the case of more than two alleles present in the cell or when FII and FIC alleles overlap. Long-read sequencing is also the only methodology capable of distinguishing multiple IncF plasmids in a single cell.

### Most common combinations of the multiple allele variants

**Fig 5:**
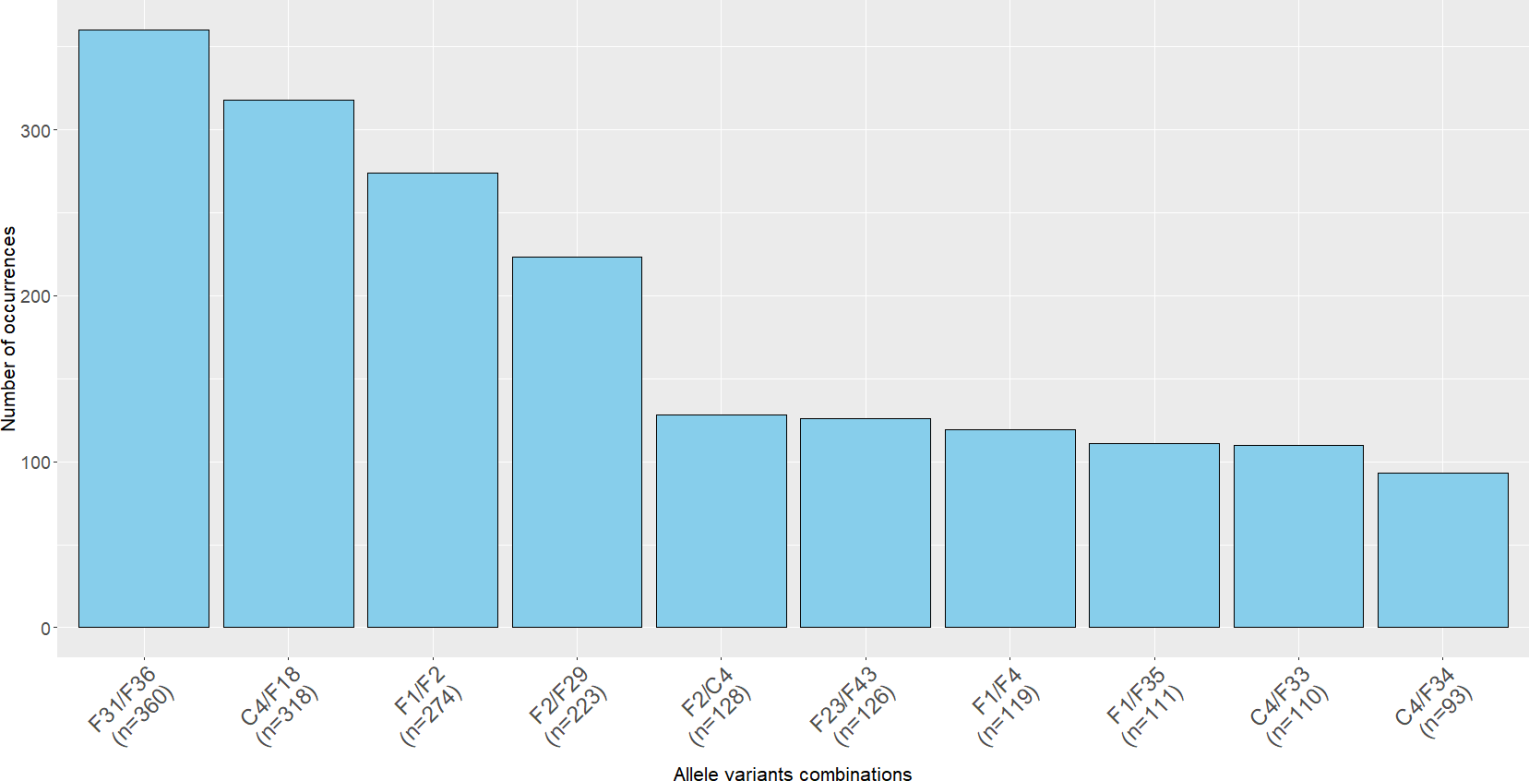
The most common allele variant combinations obtained by short-read sequencing of the complete collection of 5804 analyzed *Enterobacteriaceae* genomes carrying the multiple allele variants

From the collection of 72,469 *E. coli* and other *Enterobacteriaceae* genomes assessed here, 8.01% (*n* = 5,804) carried more than one allele variant of an IncF replicon (Table S1). Even though the phenomenon of multiple allele variants was linked mainly to the FII replicon (99.00%; 5,746/5,804), it was sporadically observed also in FIA (0.90%; 52/5,804) and FIB replicons (0.40%; 23/5,804). A small number of genomes (0.29%; 17/5,804) simultaneously carried multiple allele variants, usually in the FII allele and then either in the FIA or FIB. The number of various allele variant combinations reached up to 900. The most common combinations observed in the short-read assemblies of the whole collection were F31/F36 (6.20%; 360/5,804), C4/F18 (5.48%; 318/5,804), F1/F2 (4.72%; 274/5,804), and F2/F29 (3.84%; 223/5,804) (Fig. 5). Mistaking some of these alleles for their counterparts might obscure epidemiologically important insights. This was notable for the C4/F18 and F2/F29 allele combinations. The F18 allele often occurs in plasmids of the ColV group that are known to carry substantial and clinically important virulence gene cargo, and which are also associated with avian pathogenic *E. coli* clones in poultry (14–16). The F29 allele constitutes one part of the F29:A-:B10 FAB formulae commonly found in pUTI89-like plasmids (14, 15, 17, 18), which carry conserved *cjrABC-senB* virulence operon. This plasmid is commonly linked to *E. coli* strains causing urinary tract infections, often with nosocomial potential (17, 18). Improper pMLST reporting might lead to omission of important epidemiological insights regarding these and other strains and associated virulence and/or resistance plasmids. This was evident in our study of *E. coli* in wastewater which contained large numbers of ST131 isolates (19). The study described an FAB formula for all IncF plasmids present in the isolates; however, after rerunning the CGE pMLST tool, we found out that 21.33% (16/75) of the original FAB formulae failed to call a second IncF allele.

This phenomenon has not been described before except for a few papers where the multiple allele variants appear in the output but are not commented on further (20, 21). It was also previously reported in various *E. coli* STs, such as ST10, ST58, ST155, ST963, ST973, and ST1140, as well as in the ST we focused on the most, ST131 (20). The improper allele assignment in these cases regarded only the FII allele, which was also the most common incorrectly assigned allele in our collections. Even though various combinations were present, the F29 allele variant was the most prevalent one occurring in such cases, which highlights the importance of proper assignment due to its association with clinical human infections. Combinations of other variants were observed in a lower number of cases, none of them corresponding to the most prevalent combinations in our results (20, 21).

Our findings were supported by analyzing the prevalence of multiple allele variants carried on a single plasmid in the collection of 7,441 complete sequences of IncF plasmids obtained from GenBank. Here, the prevalence of multiple alleles was distinctly lower (3.52%, 262/7,441), in accordance with the long-read sequencing results obtained in this study. In such instances, the FAB formula from short-read analysis suggested all the replicons are located on one plasmid, but the long-read data indicated the presence of two IncF plasmids in the same cell in some instances (see Fig. 2).

### Conclusion

This study identified discrepancies between results reported by all versions of the CGE *in silico* pMLST tools compared with the biological situation regarding multiple plasmid IncF allele variants in *Enterobacteriaceae*. The CGE pMLST tool struggled to correctly designate ST and IncF alleles, particularly when multiple contigs were considered. Even though the CGE web-based tool provided us with all the IncF allele variants present in the cell, there was no possibility of assigning them to their respective plasmids unless the assembly was generated using long reads, and each putative plasmid contig was analyzed individually using the tool. The web-based output significantly differed from the Python tool regardless of the run method (Docker/Anaconda) when assessing multiple copies of the same IncF alleles in a genome assembly. Moreover, the Python-based pMLST tool was unable to detect multiple variants of IncF alleles in various cases. There was no difference in the Python tool output regardless of whether using it in a container (Docker) or installing it via Anaconda. However, in instances of multiple FII alleles, whichever allele is reported first in the table output is the one that is used in the ST designation. FAB formulae were also created by a random selection of these IncF alleles, excluding the presence of multiple variants, and in situations where there was an FII and FIC allele (both FII family), the tool completely omitted reporting the FIC allele in the formula. Since the STs of plasmids are often linked to specific bacterial lineages, inaccurate calls by the pMLST analysis report might lead to the omission of certain epidemiologically important associations.

## Data Availability

The data sets used for this study are described in the Table S1 together with corresponding Bioproject numbers.

## SUPPLEMENTAL MATERIAL

Data S1 msystems.01010-24-s0001.pdf Full output of three pMLST tool variants displaying the F31/F36 situation (Fig. 1).

Data S2 msystems.01010-24-s0002.pdf Full output of three pMLST tool variants displaying the F1/F2 situation (Fig. 2).

Data S3 msystems.01010-24-s0003.pdf Full output of three pMLST tool variants displaying the C4/F18 and B1/B58 situations (Fig. 3 and 4).

Table S1 msystems.01010-24-s0004.xlsx Metadata table displaying year, source, and country of origin of all genomes, including tool versions and bioproject numbers.

ASM does not own the copyrights to Supplemental Material that may be linked to, or accessed through, an article. The authors have granted ASM a non-exclusive, world-wide license to publish the Supplemental Material files. Please contact the corresponding author directly for reuse.
