# Supplementary material for: The presence of multiple variants of IncF plasmid alleles in a single genome sequence can hinder accurate replicon sequence typing using in silico pMLST tools
Source: mSystems. 2025 Apr 8;10(5):e01010-24. doi: 10.1128/msystems.01010-24 (PMC12090814; doi:10.1128/msystems.01010-24)
Supplement: Data S1 — Full output of three pMLST tool variants displaying the F31/F36 situation (Fig. 1). [file msystems.01010-24-s0001.pdf]

Supplement data S1. Full output of three pMLST tool variants displaying the F31/F36 situation (Fig. 1)

<u>Conda version</u>

Short-read sequence

(B0009108_Illumina.fasta)

```
pMLST profile: IncF RST

Sequence Type: [F31:A4:B1]
**********************************************************************
Locus     Identity    Coverage    Alignment Length    Allele Length
Gaps    Allele
**********************************************************************
FIA       100.0       100.0       384                 384
0       FIA_4
FIB       100.0       100.0       373                 373
0       FIB_1
FIC        -           -           -                   -
-       No hit found
FII       100.0       100.0       157                 157
0       FII_31!
FII       100.0       100.0       157                 157
0       FII_36!
FIIK       -           -           -                   -
-       No hit found
FIIS       -           -           -                   -
-       No hit found
FIIY       -           -           -                   -
-       No hit found
======================================================================

Notes: ! alleles with multiple perfect hits found, multiple STs might
be found
! FII: Multiple perfect hits found


Extended Output:

# FIA_4
template: CCATGCTGGTTCTAGAGAAGGTGTTGTGACAAATTGCCCTTAACCCTGTGACAAATTGCC
          ||||||||||||||||||||||||||||||||||||||||||||||||||||||||||||
query:    CCATGCTGGTTCTAGAGAAGGTGTTGTGACAAATTGCCCTTAACCCTGTGACAAATTGCC

template: CTCAGAAGAAGCTGTTTTTTCGCAAAGTTATCCCTGCTTATTGACTCTTTTTTATTTAGT
          ||||||||||||||||||||||||||||||||||||||||||||||||||||||||||||
query:    CTCAGAAGAAGCTGTTTTTTCGCAAAGTTATCCCTGCTTATTGACTCTTTTTTATTTAGT

template: GTGACAATCTAAAAACTTGTCACACTTCACATGGATCTGTCATGGCGGAAACAGCGGTTA
          ||||||||||||||||||||||||||||||||||||||||||||||||||||||||||||
query:    GTGACAATCTAAAAACTTGTCACACTTCACATGGATCTGTCATGGCGGAAACAGCGGTTA

template: TCAATCACAAGAAACGTAAAAATAGCCCGCGAATCGTCCAGTCAAACGACCTCACTGAGG
          ||||||||||||||||||||||||||||||||||||||||||||||||||||||||||||
query:    TCAATCACAAGAAACGTAAAAATAGCCCGCGAATCGTCCAGTCAAACGACCTCACTGAGG

template: CGGCATATAGTCTCTCCCGGGATCAAAAACGTATGCTGTATCTGTTCGTTGACCAGATCA
          ||||||||||||||||||||||||||||||||||||||||||||||||||||||||||||
query:    CGGCATATAGTCTCTCCCGGGATCAAAAACGTATGCTGTATCTGTTCGTTGACCAGATCA

template: GAAAATCTGATGGCACCCTACAGGAACATGACGGTATCTGCGAGATCCATGTTGCTAAAT
          ||||||||||||||||||||||||||||||||||||||||||||||||||||||||||||
query:    GAAAATCTGATGGCACCCTACAGGAACATGACGGTATCTGCGAGATCCATGTTGCTAAAT

template: ATGCTGAAATATTCGGATTGACCT
          ||||||||||||||||||||||||
query:    ATGCTGAAATATTCGGATTGACCT

# FII_31
template: CAAAAACCCCGATAACCTTCACCAGGTTTGGCGACTAAGAGAAGATTACCGGGGCCCACT
          ||||||||||||||||||||||||||||||||||||||||||||||||||||||||||||
query:    CAAAAACCCCGATAACCTTCACCAGGTTTGGCGACTAAGAGAAGATTACCGGGGCCCACT

template: TAAACCGTATAGCCAACAATTCAGCTATGCGGGGAGTATAGTTATATGCCCGGAAAAGTT
          ||||||||||||||||||||||||||||||||||||||||||||||||||||||||||||
query:    TAAACCGTATAGCCAACAATTCAGCTATGCGGGGAGTATAGTTATATGCCCGGAAAAGTT

template: CAAGACTTCTTTCTGTGCTCACTCCTTCTGCGCATTG
          |||||||||||||||||||||||||||||||||||||
query:    CAAGACTTCTTTCTGTGCTCACTCCTTCTGCGCATTG

# FII_36
template: CAAAAACCCCGATAATCTTCACCAGGTTTGGCGACTAAGAGAAGATTACCGGGGCCCACT
          ||||||||||||||||||||||||||||||||||||||||||||||||||||||||||||
query:    CAAAAACCCCGATAATCTTCACCAGGTTTGGCGACTAAGAGAAGATTACCGGGGCCCACT

template: TAAACCGTATAGCCAACAATTCAGCTATGCGGGGAGTATAGTTATATGCCCGGAAAAGTT
          ||||||||||||||||||||||||||||||||||||||||||||||||||||||||||||
query:    TAAACCGTATAGCCAACAATTCAGCTATGCGGGGAGTATAGTTATATGCCCGGAAAAGTT

template: CAAGACTTCTTTCTGTGCTCACTCCTTCTGCGCATTG
          |||||||||||||||||||||||||||||||||||||
query:    CAAGACTTCTTTCTGTGCTCACTCCTTCTGCGCATTG

# FIB_1
template: ATTCAGACATCAAAAAACTGTTCGGCGAGGTGGATAAGTCCTCCGGTGAGCTGGTGACAC
          ||||||||||||||||||||||||||||||||||||||||||||||||||||||||||||
query:    ATTCAGACATCAAAAAACTGTTCGGCGAGGTGGATAAGTCCTCCGGTGAGCTGGTGACAC

template: TGACACCAAACAATAACAACACCGTACAACCTGTGGCGCTGATGCGTCTGGGCGTTTTTG
          ||||||||||||||||||||||||||||||||||||||||||||||||||||||||||||
query:    TGACACCAAACAATAACAACACCGTACAACCTGTGGCGCTGATGCGTCTGGGCGTTTTTG

template: TACCGACCCTTAAATCACTGAAGAACAGTAAAAAAAAATACACTGTCACGTACTGATGCCA
          ||||||||||||||||||||||||||||||||||||||||||||||||||||||||||||
query:    TACCGACCCTTAAATCACTGAAGAACAGTAAAAAAAAATACACTGTCACGTACTGATGCCA

template: CGGAAGAGCTGACACGTCTTTCCCTGGCCCGTGCTGAGGGATTCGATAAGGTTGAGATCA
          ||||||||||||||||||||||||||||||||||||||||||||||||||||||||||||
query:    CGGAAGAGCTGACACGTCTTTCCCTGGCCCGTGCTGAGGGATTCGATAAGGTTGAGATCA

template: CCGGCCCCCGCCTGGATATGGATAATGATTTCAAGACCTGGGTGGGGATCATTCATTCCT
          ||||||||||||||||||||||||||||||||||||||||||||||||||||||||||||
query:    CCGGCCCCCGCCTGGATATGGATAATGATTTCAAGACCTGGGTGGGGATCATTCATTCCT

template: TTGCCCGCCATAACGTGATTGGTGACAAAGTTGAACTGCCTTTTGTTGAGTTTGCAAAAC
          ||||||||||||||||||||||||||||||||||||||||||||||||||||||||||||
query:    TTGCCCGCCATAACGTGATTGGTGACAAAGTTGAACTGCCTTTTGTTGAGTTTGCAAAAC

template: TGTGTGGTATACC
          |||||||||||||
query:    TGTGTGGTATACC
```

Conda version

Long-read sequence

(B0009108_F31-F36_A4_B1_minion.fasta)

```
pMLST profile: IncF RST

Sequence Type: [F36:A4:B58]
*********************************************************************
Locus    Identity    Coverage           Alignment Length    Allele
Length    Gaps    Allele
*********************************************************************
FIA      100.0       100.0              384                 384
0       FIA_4
FIB      100.0       92.22520107238606  344                 373
0       FIB_58?
FIC      -           -                  -                   -
-       No hit found
FII      100.0       100.0              157                 157
0       FII_36!
FII      100.0       100.0              157                 157
0       FII_31!
FIIK     -           -                  -                   -
-       No hit found
FIIS     -           -                  -                   -
-       No hit found
FIIY     -           -                  -                   -
-       No hit found
=====================================================================

Notes: !? alleles with multiple perfect hits found, multiple STs might
be found
? FIB: Uncertain hit, ST can not be trusted.
! FII: Multiple perfect hits found


Extended Output:

# FIA_4
template: CCATGCTGGTTCTAGAGAAGGTGTTGTGACAAATTGCCCTTAACCCTGTGACAAATTGCC
          ||||||||||||||||||||||||||||||||||||||||||||||||||||||||||||
query:    CCATGCTGGTTCTAGAGAAGGTGTTGTGACAAATTGCCCTTAACCCTGTGACAAATTGCC

template: CTCAGAAGAAGCTGTTTTTTCGCAAAGTTATCCCTGCTTATTGACTCTTTTTTATTTAGT
          ||||||||||||||||||||||||||||||||||||||||||||||||||||||||||||
query:    CTCAGAAGAAGCTGTTTTTTCGCAAAGTTATCCCTGCTTATTGACTCTTTTTTATTTAGT

template: GTGACAATCTAAAAACTTGTCACACTTCACATGGATCTGTCATGGCGGAAACAGCGGTTA
          ||||||||||||||||||||||||||||||||||||||||||||||||||||||||||||
query:    GTGACAATCTAAAAACTTGTCACACTTCACATGGATCTGTCATGGCGGAAACAGCGGTTA

template: TCAATCACAAGAAACGTAAAAATAGCCCGCGAATCGTCCAGTCAAACGACCTCACTGAGG
          ||||||||||||||||||||||||||||||||||||||||||||||||||||||||||||
query:    TCAATCACAAGAAACGTAAAAATAGCCCGCGAATCGTCCAGTCAAACGACCTCACTGAGG

template: CGGCATATAGTCTCTCCCGGGATCAAAAACGTATGCTGTATCTGTTCGTTGACCAGATCA
          ||||||||||||||||||||||||||||||||||||||||||||||||||||||||||||
query:    CGGCATATAGTCTCTCCCGGGATCAAAAACGTATGCTGTATCTGTTCGTTGACCAGATCA

template: GAAAATCTGATGGCACCCTACAGGAACATGACGGTATCTGCGAGATCCATGTTGCTAAAT
          ||||||||||||||||||||||||||||||||||||||||||||||||||||||||||||
query:    GAAAATCTGATGGCACCCTACAGGAACATGACGGTATCTGCGAGATCCATGTTGCTAAAT

template: ATGCTGAAATATTCGGATTGACCT
          ||||||||||||||||||||||||
query:    ATGCTGAAATATTCGGATTGACCT

# FIB_58
template: ATTCAGACATAAAAAAACTGTTCGGCGAGGTGGATAAGTCCTCCGGTGAGCTGGTGACAC
                                       |||||||||||||||||||||||||||||||
query:    -----------------------------GTGGATAAGTCCTCCGGTGAGCTGGTGACAC

template: TGACACCAAACAATAACAACACCGTACAACCTGTGGCGCTGATGCGTCTGGGCGTTTTTG
          ||||||||||||||||||||||||||||||||||||||||||||||||||||||||||||
query:    TGACACCAAACAATAACAACACCGTACAACCTGTGGCGCTGATGCGTCTGGGCGTTTTTG

template: TACCGACCCTTAAATCACTGAAGAACAGTAAAAAAAAATACACTGTCACGTACTGATGCCA
          ||||||||||||||||||||||||||||||||||||||||||||||||||||||||||||
query:    TACCGACCCTTAAATCACTGAAGAACAGTAAAAAAAAATACACTGTCACGTACTGATGCCA

template: CGGAAGAGCTGACACGTCTTTCCCTGGCCCGTGCTGAGGGATTCGATAAGGTTGAGATCA
          ||||||||||||||||||||||||||||||||||||||||||||||||||||||||||||
query:    CGGAAGAGCTGACACGTCTTTCCCTGGCCCGTGCTGAGGGATTCGATAAGGTTGAGATCA

template: CCGGCCCCCGCCTGGATATGGATAATGATTTCAAGACCTGGGTGGGGATCATTCATTCCT
          ||||||||||||||||||||||||||||||||||||||||||||||||||||||||||||
query:    CCGGCCCCCGCCTGGATATGGATAATGATTTCAAGACCTGGGTGGGGATCATTCATTCCT

template: TTGCCCGCCATAACGTGATTGGTGACAAAGTTGAACTGCCTTTTGTTGAGTTTGCAAAAC
          ||||||||||||||||||||||||||||||||||||||||||||||||||||||||||||
query:    TTGCCCGCCATAACGTGATTGGTGACAAAGTTGAACTGCCTTTTGTTGAGTTTGCAAAAC

template: TGTGTGGTATACC
          |||||||||||||
query:    TGTGTGGTATACC

# FII_36
template: CAAAAACCCCGATAATCTTCACCAGGTTTGGCGACTAAGAGAAGATTACCGGGGCCCACT
          ||||||||||||||||||||||||||||||||||||||||||||||||||||||||||||
query:    CAAAAACCCCGATAATCTTCACCAGGTTTGGCGACTAAGAGAAGATTACCGGGGCCCACT

template: TAAACCGTATAGCCAACAATTCAGCTATGCGGGGAGTATAGTTATATGCCCGGAAAAGTT
          ||||||||||||||||||||||||||||||||||||||||||||||||||||||||||||
query:    TAAACCGTATAGCCAACAATTCAGCTATGCGGGGAGTATAGTTATATGCCCGGAAAAGTT

template: CAAGACTTCTTTCTGTGCTCACTCCTTCTGCGCATTG
          |||||||||||||||||||||||||||||||||||||
query:    CAAGACTTCTTTCTGTGCTCACTCCTTCTGCGCATTG

# FII_31
template: CAAAAACCCCGATAACCTTCACCAGGTTTGGCGACTAAGAGAAGATTACCGGGGCCCACT
          ||||||||||||||||||||||||||||||||||||||||||||||||||||||||||||
query:    CAAAAACCCCGATAACCTTCACCAGGTTTGGCGACTAAGAGAAGATTACCGGGGCCCACT

template: TAAACCGTATAGCCAACAATTCAGCTATGCGGGGAGTATAGTTATATGCCCGGAAAAGTT
          ||||||||||||||||||||||||||||||||||||||||||||||||||||||||||||
query:    TAAACCGTATAGCCAACAATTCAGCTATGCGGGGAGTATAGTTATATGCCCGGAAAAGTT

template: CAAGACTTCTTTCTGTGCTCACTCCTTCTGCGCATTG
          |||||||||||||||||||||||||||||||||||||
query:    CAAGACTTCTTTCTGTGCTCACTCCTTCTGCGCATTG
```

<u>**Docker version**</u>

Short-read sequence

(B0009108_Illumina.fasta)

```
pMLST profile: IncF RST

Sequence Type: [F31:A4:B1]
**********************************************************************
Locus    Identity    Coverage    Alignment Length    Allele Length
Gaps    Allele
**********************************************************************
FIA      100.0       100.0       384                 384
0       FIA_4
FIB      100.0       100.0       373                 373
0       FIB_1
FIC      -           -           -                   -
-       No hit found
FII      100.0       100.0       157                 157
0       FII_31!
FII      100.0       100.0       157                 157
0       FII_36!
FIIK     -           -           -                   -
-       No hit found
FIIS     -           -           -                   -
-       No hit found
FIIY     -           -           -                   -
-       No hit found
======================================================================

Notes: ! alleles with multiple perfect hits found, multiple STs might
be found
! FII: Multiple perfect hits found


Extended Output:

# FII_31
template: CAAAAACCCCGATAACCTTCACCAGGTTTGGCGACTAAGAGAAGATTACCGGGGCCCACT
          ||||||||||||||||||||||||||||||||||||||||||||||||||||||||||||
query:    CAAAAACCCCGATAACCTTCACCAGGTTTGGCGACTAAGAGAAGATTACCGGGGCCCACT

template: TAAACCGTATAGCCAACAATTCAGCTATGCGGGGAGTATAGTTATATGCCCGGAAAAGTT
          ||||||||||||||||||||||||||||||||||||||||||||||||||||||||||||
query:    TAAACCGTATAGCCAACAATTCAGCTATGCGGGGAGTATAGTTATATGCCCGGAAAAGTT

template: CAAGACTTCTTTCTGTGCTCACTCCTTCTGCGCATTG
          |||||||||||||||||||||||||||||||||||||
query:    CAAGACTTCTTTCTGTGCTCACTCCTTCTGCGCATTG

# FII_36
template: CAAAAACCCCGATAATCTTCACCAGGTTTGGCGACTAAGAGAAGATTACCGGGGCCCACT
          ||||||||||||||||||||||||||||||||||||||||||||||||||||||||||||
query:    CAAAAACCCCGATAATCTTCACCAGGTTTGGCGACTAAGAGAAGATTACCGGGGCCCACT

template: TAAACCGTATAGCCAACAATTCAGCTATGCGGGGAGTATAGTTATATGCCCGGAAAAGTT
          ||||||||||||||||||||||||||||||||||||||||||||||||||||||||||||
query:    TAAACCGTATAGCCAACAATTCAGCTATGCGGGGAGTATAGTTATATGCCCGGAAAAGTT

template: CAAGACTTCTTTCTGTGCTCACTCCTTCTGCGCATTG
          |||||||||||||||||||||||||||||||||||||
query:    CAAGACTTCTTTCTGTGCTCACTCCTTCTGCGCATTG

# FIB_1
template: ATTCAGACATCAAAAAACTGTTCGGCGAGGTGGATAAGTCCTCCGGTGAGCTGGTGACAC
          ||||||||||||||||||||||||||||||||||||||||||||||||||||||||||||
query:    ATTCAGACATCAAAAAACTGTTCGGCGAGGTGGATAAGTCCTCCGGTGAGCTGGTGACAC

template: TGACACCAAACAATAACAACACCGTACAACCTGTGGCGCTGATGCGTCTGGGCGTTTTTG
          ||||||||||||||||||||||||||||||||||||||||||||||||||||||||||||
query:    TGACACCAAACAATAACAACACCGTACAACCTGTGGCGCTGATGCGTCTGGGCGTTTTTG

template: TACCGACCCTTAAATCACTGAAGAACAGTAAAAAAAAATACACTGTCACGTACTGATGCCA
          ||||||||||||||||||||||||||||||||||||||||||||||||||||||||||||
query:    TACCGACCCTTAAATCACTGAAGAACAGTAAAAAAAAATACACTGTCACGTACTGATGCCA

template: CGGAAGAGCTGACACGTCTTTCCCTGGCCCGTGCTGAGGGATTCGATAAGGTTGAGATCA
          ||||||||||||||||||||||||||||||||||||||||||||||||||||||||||||
query:    CGGAAGAGCTGACACGTCTTTCCCTGGCCCGTGCTGAGGGATTCGATAAGGTTGAGATCA

template: CCGGCCCCCGCCTGGATATGGATAATGATTTCAAGACCTGGGTGGGGATCATTCATTCCT
          ||||||||||||||||||||||||||||||||||||||||||||||||||||||||||||
query:    CCGGCCCCCGCCTGGATATGGATAATGATTTCAAGACCTGGGTGGGGATCATTCATTCCT

template: TTGCCCGCCATAACGTGATTGGTGACAAAGTTGAACTGCCTTTTGTTGAGTTTGCAAAAC
          ||||||||||||||||||||||||||||||||||||||||||||||||||||||||||||
query:    TTGCCCGCCATAACGTGATTGGTGACAAAGTTGAACTGCCTTTTGTTGAGTTTGCAAAAC

template: TGTGTGGTATACC
          |||||||||||||
query:    TGTGTGGTATACC

# FIA_4
template: CCATGCTGGTTCTAGAGAAGGTGTTGTGACAAATTGCCCTTAACCCTGTGACAAATTGCC
          ||||||||||||||||||||||||||||||||||||||||||||||||||||||||||||
query:    CCATGCTGGTTCTAGAGAAGGTGTTGTGACAAATTGCCCTTAACCCTGTGACAAATTGCC

template: CTCAGAAGAAGCTGTTTTTTCGCAAAGTTATCCCTGCTTATTGACTCTTTTTTATTTAGT
          ||||||||||||||||||||||||||||||||||||||||||||||||||||||||||||
query:    CTCAGAAGAAGCTGTTTTTTCGCAAAGTTATCCCTGCTTATTGACTCTTTTTTATTTAGT

template: GTGACAATCTAAAAACTTGTCACACTTCACATGGATCTGTCATGGCGGAAACAGCGGTTA
          ||||||||||||||||||||||||||||||||||||||||||||||||||||||||||||
query:    GTGACAATCTAAAAACTTGTCACACTTCACATGGATCTGTCATGGCGGAAACAGCGGTTA

template: TCAATCACAAGAAACGTAAAAATAGCCCGCGAATCGTCCAGTCAAACGACCTCACTGAGG
          ||||||||||||||||||||||||||||||||||||||||||||||||||||||||||||
query:    TCAATCACAAGAAACGTAAAAATAGCCCGCGAATCGTCCAGTCAAACGACCTCACTGAGG

template: CGGCATATAGTCTCTCCCGGGATCAAAAACGTATGCTGTATCTGTTCGTTGACCAGATCA
          ||||||||||||||||||||||||||||||||||||||||||||||||||||||||||||
query:    CGGCATATAGTCTCTCCCGGGATCAAAAACGTATGCTGTATCTGTTCGTTGACCAGATCA

template: GAAAATCTGATGGCACCCTACAGGAACATGACGGTATCTGCGAGATCCATGTTGCTAAAT
          ||||||||||||||||||||||||||||||||||||||||||||||||||||||||||||
query:    GAAAATCTGATGGCACCCTACAGGAACATGACGGTATCTGCGAGATCCATGTTGCTAAAT

template: ATGCTGAAATATTCGGATTGACCT
          ||||||||||||||||||||||||
query:    ATGCTGAAATATTCGGATTGACCT
```

Docker version

Long-read sequence

(B0009108_F31-F36_A4_B1_minion.fasta)

```
pMLST profile: IncF RST

Sequence Type: [F36:A4:B1]
**********************************************************************
Locus    Identity    Coverage           Alignment Length    Allele
Length    Gaps    Allele
**********************************************************************
FIA       100.0       100.0              384                 384
0       FIA_4
FIB       100.0       92.22520107238606  344                 373
0       FIB_1?
FIC       -           -                  -                   -
-       No hit found
FII       100.0       100.0              157                 157
0       FII_36!
FII       100.0       100.0              157                 157
0       FII_31!
FIIK      -           -                  -                   -
-       No hit found
FIIS      -           -                  -                   -
-       No hit found
FIIY      -           -                  -                   -
-       No hit found
======================================================================

Notes: ?! alleles with multiple perfect hits found, multiple STs might
be found
! FII: Multiple perfect hits found
? FIB: Uncertain hit, ST can not be trusted.


Extended Output:

# FII_36
template: CAAAAACCCCGATAATCTTCACCAGGTTTGGCGACTAAGAGAAGATTACCGGGGCCCACT
          ||||||||||||||||||||||||||||||||||||||||||||||||||||||||||||
query:    CAAAAACCCCGATAATCTTCACCAGGTTTGGCGACTAAGAGAAGATTACCGGGGCCCACT

template: TAAACCGTATAGCCAACAATTCAGCTATGCGGGGAGTATAGTTATATGCCCGGAAAAGTT
          ||||||||||||||||||||||||||||||||||||||||||||||||||||||||||||
query:    TAAACCGTATAGCCAACAATTCAGCTATGCGGGGAGTATAGTTATATGCCCGGAAAAGTT

template: CAAGACTTCTTTCTGTGCTCACTCCTTCTGCGCATTG
          |||||||||||||||||||||||||||||||||||||
query:    CAAGACTTCTTTCTGTGCTCACTCCTTCTGCGCATTG

# FII_31
template: CAAAAACCCCGATAACCTTCACCAGGTTTGGCGACTAAGAGAAGATTACCGGGGCCCACT
          ||||||||||||||||||||||||||||||||||||||||||||||||||||||||||||
query:    CAAAAACCCCGATAACCTTCACCAGGTTTGGCGACTAAGAGAAGATTACCGGGGCCCACT

template: TAAACCGTATAGCCAACAATTCAGCTATGCGGGGAGTATAGTTATATGCCCGGAAAAGTT
          ||||||||||||||||||||||||||||||||||||||||||||||||||||||||||||
query:    TAAACCGTATAGCCAACAATTCAGCTATGCGGGGAGTATAGTTATATGCCCGGAAAAGTT

template: CAAGACTTCTTTCTGTGCTCACTCCTTCTGCGCATTG
          |||||||||||||||||||||||||||||||||||||
query:    CAAGACTTCTTTCTGTGCTCACTCCTTCTGCGCATTG

# FIB_1
template: ATTCAGACATCAAAAAACTGTTCGGCGAGGTGGATAAGTCCTCCGGTGAGCTGGTGACAC
                                       |||||||||||||||||||||||||||||||
query:    -----------------------------GTGGATAAGTCCTCCGGTGAGCTGGTGACAC

template: TGACACCAAACAATAACAACACCGTACAACCTGTGGCGCTGATGCGTCTGGGCGTTTTTG
          ||||||||||||||||||||||||||||||||||||||||||||||||||||||||||||
query:    TGACACCAAACAATAACAACACCGTACAACCTGTGGCGCTGATGCGTCTGGGCGTTTTTG

template: TACCGACCCTTAAATCACTGAAGAACAGTAAAAAAAATACACTGTCACGTACTGATGCCA
          ||||||||||||||||||||||||||||||||||||||||||||||||||||||||||||
query:    TACCGACCCTTAAATCACTGAAGAACAGTAAAAAAAATACACTGTCACGTACTGATGCCA

template: CGGAAGAGCTGACACGTCTTTCCCTGGCCCGTGCTGAGGGATTCGATAAGGTTGAGATCA
          ||||||||||||||||||||||||||||||||||||||||||||||||||||||||||||
query:    CGGAAGAGCTGACACGTCTTTCCCTGGCCCGTGCTGAGGGATTCGATAAGGTTGAGATCA

template: CCGGCCCCCGCCTGGATATGGATAATGATTTCAAGACCTGGGTGGGGATCATTCATTCCT
          ||||||||||||||||||||||||||||||||||||||||||||||||||||||||||||
query:    CCGGCCCCCGCCTGGATATGGATAATGATTTCAAGACCTGGGTGGGGATCATTCATTCCT

template: TTGCCCGCCATAACGTGATTGGTGACAAAGTTGAACTGCCTTTTGTTGAGTTTGCAAAAC
          ||||||||||||||||||||||||||||||||||||||||||||||||||||||||||||
query:    TTGCCCGCCATAACGTGATTGGTGACAAAGTTGAACTGCCTTTTGTTGAGTTTGCAAAAC

template: TGTGTGGTATACC
          |||||||||||||
query:    TGTGTGGTATACC

# FIA_4
template: CCATGCTGGTTCTAGAGAAGGTGTTGTGACAAATTGCCCTTAACCCTGTGACAAATTGCC
          ||||||||||||||||||||||||||||||||||||||||||||||||||||||||||||
query:    CCATGCTGGTTCTAGAGAAGGTGTTGTGACAAATTGCCCTTAACCCTGTGACAAATTGCC

template: CTCAGAAGAAGCTGTTTTTTCGCAAAGTTATCCCTGCTTATTGACTCTTTTTTATTTAGT
          ||||||||||||||||||||||||||||||||||||||||||||||||||||||||||||
query:    CTCAGAAGAAGCTGTTTTTTCGCAAAGTTATCCCTGCTTATTGACTCTTTTTTATTTAGT

template: GTGACAATCTAAAAACTTGTCACACTTCACATGGATCTGTCATGGCGGAAACAGCGGTTA
          ||||||||||||||||||||||||||||||||||||||||||||||||||||||||||||
query:    GTGACAATCTAAAAACTTGTCACACTTCACATGGATCTGTCATGGCGGAAACAGCGGTTA

template: TCAATCACAAGAAACGTAAAAATAGCCCGCGAATCGTCCAGTCAAACGACCTCACTGAGG
          ||||||||||||||||||||||||||||||||||||||||||||||||||||||||||||
query:    TCAATCACAAGAAACGTAAAAATAGCCCGCGAATCGTCCAGTCAAACGACCTCACTGAGG

template: CGGCATATAGTCTCTCCCGGGATCAAAAACGTATGCTGTATCTGTTCGTTGACCAGATCA
          ||||||||||||||||||||||||||||||||||||||||||||||||||||||||||||
query:    CGGCATATAGTCTCTCCCGGGATCAAAAACGTATGCTGTATCTGTTCGTTGACCAGATCA

template: GAAAATCTGATGGCACCCTACAGGAACATGACGGTATCTGCGAGATCCATGTTGCTAAAT
          ||||||||||||||||||||||||||||||||||||||||||||||||||||||||||||
query:    GAAAATCTGATGGCACCCTACAGGAACATGACGGTATCTGCGAGATCCATGTTGCTAAAT

template: ATGCTGAAATATTCGGATTGACCT
          ||||||||||||||||||||||||
query:    ATGCTGAAATATTCGGATTGACCT
```

CGE web version

Short-read sequence

(B0009108_Illumina.fasta)

1st run

pMLST profile: *IncF RST*

Sequence Type: *[F31:A4:B1]*

Locus	Identity	Coverage	Alignment Length	Allele Length	Gaps	Allele
FIA	100.0	100.0	384	384	0	FIA_4
FIB	100.0	100.0	373	373	0	FIB_1
FIC	98.61111111111111	72.0	144	200	0	FIC_3?*
FII	100.0	100.0	157	157	0	FII_31!
FII	100.0	100.0	157	157	0	FII_36!
FIIK						No hit found
FIIS						No hit found
FIIY						No hit found

Notes: *?! alleles with multiple perfect hits found, multiple STs might be found

?* FIC: *Imperfect hit, ST can not be trusted!*

! FII: *Multiple perfect hits found*

2nd run

pMLST profile: *IncF RST*

Sequence Type: *[F36:A4:B1]*

Locus	Identity	Coverage	Alignment Length	Allele Length	Gaps	Allele
FIA	100.0	100.0	384	384	0	FIA_4
FIB	100.0	100.0	373	373	0	FIB_1
FIC	98.61111111111111	72.0	144	200	0	FIC_3?*
FII	100.0	100.0	157	157	0	FII_36!
FII	100.0	100.0	157	157	0	FII_31!
FIIK						No hit found
FIIS						No hit found
FIIY						No hit found

Notes: *? alleles with less than 100% identity and 100% coverages found

! FII: *Multiple perfect hits found*

?* FIC: *Imperfect hit, ST can not be trusted!*

CGE web version

Long-read sequence

(B0009108_F31-F36_A4_B1_minion.fasta)

pMLST profile: *IncF RST*

Sequence Type: *[F36:A4:B58]*

Locus	Identity	Coverage	Alignment Length	Allele Length	Gaps	Allele
FIA	100.0	100.0	384	384	0	FIA_4
FIB	100.0	92.22520107238606	344	373	0	FIB_58?
FIC	98.61111111111111	72.0	144	200	0	FIC_3?*
FII	100.0	100.0	157	157	0	FII_36!
FII	100.0	100.0	157	157	0	FII_31!
FIIK						No hit found
FIIS						No hit found
FIIY						No hit found

Notes: !?* alleles with multiple perfect hits found, multiple STs might be found

? FIB: *Uncertain hit, ST can not be trusted.*

?* FIC: *Imperfect hit, ST can not be trusted!*

! FII: *Multiple perfect hits found*
