# Supplementary material for: The presence of multiple variants of IncF plasmid alleles in a single genome sequence can hinder accurate replicon sequence typing using in silico pMLST tools
Source: mSystems. 2025 Apr 8;10(5):e01010-24. doi: 10.1128/msystems.01010-24 (PMC12090814; doi:10.1128/msystems.01010-24)
Supplement: Data S2 — Full output of three pMLST tool variants displaying the F1/F2 situation (Fig. 2). [file msystems.01010-24-s0002.pdf]

Supplement data S2. Full output of three pMLST tool variants displaying the F1/F2 situation (Fig. 2)

Conda version

Short-read sequence

(OV_CH_97_Illumina.fasta)

```
pMLST profile: IncF RST

Sequence Type: [F2:A2:B20]
**********************************************************************
Locus    Identity    Coverage    Alignment Length    Allele Length
Gaps    Allele
**********************************************************************
FIA      100.0       100.0       384                 384
0       FIA_2
FIB      100.0       100.0       373                 373
0       FIB_20
FIC      -           -           -                   -
-       No hit found
FII      100.0       100.0       157                 157
0       FII_2!
FII      100.0       100.0       157                 157
0       FII_1!
FIIK     -           -           -                   -
-       No hit found
FIIS     -           -           -                   -
-       No hit found
FIIY     -           -           -                   -
-       No hit found
======================================================================

Notes: ! alleles with multiple perfect hits found, multiple STs might
be found
! FII: Multiple perfect hits found


Extended Output:

# FII_2
template: CAAAAACCCCGATAATCTTCTTCAACTTTGGCGAGTACGAAAAGATTACCGGGGCCCACT
          ||||||||||||||||||||||||||||||||||||||||||||||||||||||||||||
query:    CAAAAACCCCGATAATCTTCTTCAACTTTGGCGAGTACGAAAAGATTACCGGGGCCCACT

template: TAAACCGTATAGCCAACAATTCAGCTATGCGGGGAGTATAGTTATATGCCCGGAAAAGTT
          ||||||||||||||||||||||||||||||||||||||||||||||||||||||||||||
query:    TAAACCGTATAGCCAACAATTCAGCTATGCGGGGAGTATAGTTATATGCCCGGAAAAGTT

template: CAAGACTTCTTTCTGTGCTCGCTCCTTCTGCGCATTG
          |||||||||||||||||||||||||||||||||||||
query:    CAAGACTTCTTTCTGTGCTCGCTCCTTCTGCGCATTG

# FII_1
template: CAAAAACCCCGATAATCTTCACCAGGTTTGGCGACTAAGAGAAGATTACCGGGGCTAACA
          ||||||||||||||||||||||||||||||||||||||||||||||||||||||||||||
query:    CAAAAACCCCGATAATCTTCACCAGGTTTGGCGACTAAGAGAAGATTACCGGGGCTAACA

template: AGAAACTGCATAGAAGCTGTTGCTCTATGCGGGGAGTATAGTTATATGACCGGAAAAGTT
          ||||||||||||||||||||||||||||||||||||||||||||||||||||||||||||
query:    AGAAACTGCATAGAAGCTGTTGCTCTATGCGGGGAGTATAGTTATATGACCGGAAAAGTT

template: CAAGACTTCTTTCTGTGCTCACTCCTTCTGTGCAACA
          |||||||||||||||||||||||||||||||||||||
query:    CAAGACTTCTTTCTGTGCTCACTCCTTCTGTGCAACA

# FIA_2
template: TCACCCTCAAATGACAGTCCTGTCTGTGACAAATTGCCCTTAACCCTGTGACAAATTGCC
          ||||||||||||||||||||||||||||||||||||||||||||||||||||||||||||
query:    TCACCCTCAAATGACAGTCCTGTCTGTGACAAATTGCCCTTAACCCTGTGACAAATTGCC

template: CTCAGAAGAAGCTGTTTTTTCACAAAGTTATCCCTGCTTATTGACTCTTTTTTATTTAGT
          ||||||||||||||||||||||||||||||||||||||||||||||||||||||||||||
query:    CTCAGAAGAAGCTGTTTTTTCACAAAGTTATCCCTGCTTATTGACTCTTTTTTATTTAGT

template: GTGACAATCTAAAAACTTGTCACACTTCACATGGATCTGTCATGGCGGAAACAGCGGTTA
          ||||||||||||||||||||||||||||||||||||||||||||||||||||||||||||
query:    GTGACAATCTAAAAACTTGTCACACTTCACATGGATCTGTCATGGCGGAAACAGCGGTTA

template: TCAATCACAAGAAACGTAAAAATAGCCCGCGAATCGTCCAGTCAAACGACCTCACTGAGG
          ||||||||||||||||||||||||||||||||||||||||||||||||||||||||||||
query:    TCAATCACAAGAAACGTAAAAATAGCCCGCGAATCGTCCAGTCAAACGACCTCACTGAGG

template: CGGCATATAGTCTCTCCCGGGATCAAAAACGTATGCTGTATCTGTTCGTTGACCAGATCA
          ||||||||||||||||||||||||||||||||||||||||||||||||||||||||||||
query:    CGGCATATAGTCTCTCCCGGGATCAAAAACGTATGCTGTATCTGTTCGTTGACCAGATCA

template: GAAAATCTGATGGCACCCTACAGGAACATGACGGTATCTGCGAGATCCATGTTGCTAAAT
          ||||||||||||||||||||||||||||||||||||||||||||||||||||||||||||
query:    GAAAATCTGATGGCACCCTACAGGAACATGACGGTATCTGCGAGATCCATGTTGCTAAAT

template: ATGCTGAAATATTCGGATTGACCT
          ||||||||||||||||||||||||
query:    ATGCTGAAATATTCGGATTGACCT

# FIB_20
template: ATTCAGACATCAAAAAACTGTTCGGCGAGGTGGATAAGTCGTCCGGTGAGCTGGTGACAC
          ||||||||||||||||||||||||||||||||||||||||||||||||||||||||||||
query:    ATTCAGACATCAAAAAACTGTTCGGCGAGGTGGATAAGTCGTCCGGTGAGCTGGTGACAC

template: TGACACCAAACAATAACAACACCGTACAACCTGTGGCGCTGATGCGTCTGGGCGTCTTTG
          ||||||||||||||||||||||||||||||||||||||||||||||||||||||||||||
query:    TGACACCAAACAATAACAACACCGTACAACCTGTGGCGCTGATGCGTCTGGGCGTCTTTG

template: TACCGACCCTTAAGTCACTGAAGAACAGTAAAAAAAAATACACTGTCACGCACCGATGCCA
          |||||||||||||||||||||||||||||||||||||||||||||||||||||||||||||
query:    TACCGACCCTTAAGTCACTGAAGAACAGTAAAAAAAAATACACTGTCACGCACCGATGCCA

template: CGGAAGAACTGACGCGTCTTTCTCTGGCCCGTGCAGAAGGATTCGATAAGGTTGAGATCA
          ||||||||||||||||||||||||||||||||||||||||||||||||||||||||||||
query:    CGGAAGAACTGACGCGTCTTTCTCTGGCCCGTGCAGAAGGATTCGATAAGGTTGAGATCA

template: CCGGCCCCCGGCTGGATATGGATAACGATTTCAAGACCTGGGTGGGGATCATTCATTCCT
          ||||||||||||||||||||||||||||||||||||||||||||||||||||||||||||
query:    CCGGCCCCCGGCTGGATATGGATAACGATTTCAAGACCTGGGTGGGGATCATTCATTCCT

template: TTGCCCGCCATAACGTGATTGGTGACAAAGTTGAACTGCCTTTTGTTGAGTTTGCAAAAC
          ||||||||||||||||||||||||||||||||||||||||||||||||||||||||||||
query:    TTGCCCGCCATAACGTGATTGGTGACAAAGTTGAACTGCCTTTTGTTGAGTTTGCAAAAC

template: TGTGTGGTATACC
          |||||||||||||
query:    TGTGTGGTATACC
```

Conda version

Long-read sequence

(OV_CH_97_F1_A2_B20_minion.fasta)

```
pMLST profile: IncF RST

Sequence Type: [F1:A2:B20]
**********************************************************************
Locus     Identity    Coverage            Alignment Length    Allele
Length    Gaps    Allele
**********************************************************************
FIA       100.0       100.0               384                 384
0        FIA_2
FIB       100.0       92.22520107238606   344                 373
0        FIB_20?
FIC       -           -                   -                   -
-        No hit found
FII       100.0       100.0               157                 157
0        FII_1
FIIK      -           -                   -                   -
-        No hit found
FIIS      -           -                   -                   -
-        No hit found
FIIY      -           -                   -                   -
-        No hit found
======================================================================

Notes: ? alleles with less than 100% coverage found
? FIB: Uncertain hit, ST can not be trusted.


Extended Output:

# FIA_2
template: TCACCCTCAAATGACAGTCCTGTCTGTGACAAATTGCCCTTAACCCTGTGACAAATTGCC
          ||||||||||||||||||||||||||||||||||||||||||||||||||||||||||||
query:    TCACCCTCAAATGACAGTCCTGTCTGTGACAAATTGCCCTTAACCCTGTGACAAATTGCC

template: CTCAGAAGAAGCTGTTTTTTCACAAAGTTATCCCTGCTTATTGACTCTTTTTTATTTAGT
          ||||||||||||||||||||||||||||||||||||||||||||||||||||||||||||
query:    CTCAGAAGAAGCTGTTTTTTCACAAAGTTATCCCTGCTTATTGACTCTTTTTTATTTAGT

template: GTGACAATCTAAAAACTTGTCACACTTCACATGGATCTGTCATGGCGGAAACAGCGGTTA
          ||||||||||||||||||||||||||||||||||||||||||||||||||||||||||||
query:    GTGACAATCTAAAAACTTGTCACACTTCACATGGATCTGTCATGGCGGAAACAGCGGTTA

template: TCAATCACAAGAAACGTAAAAATAGCCCGCGAATCGTCCAGTCAAACGACCTCACTGAGG
          ||||||||||||||||||||||||||||||||||||||||||||||||||||||||||||
query:    TCAATCACAAGAAACGTAAAAATAGCCCGCGAATCGTCCAGTCAAACGACCTCACTGAGG

template: CGGCATATAGTCTCTCCCGGGATCAAAAACGTATGCTGTATCTGTTCGTTGACCAGATCA
          ||||||||||||||||||||||||||||||||||||||||||||||||||||||||||||
query:    CGGCATATAGTCTCTCCCGGGATCAAAAACGTATGCTGTATCTGTTCGTTGACCAGATCA

template: GAAAATCTGATGGCACCCTACAGGAACATGACGGTATCTGCGAGATCCATGTTGCTAAAT
          ||||||||||||||||||||||||||||||||||||||||||||||||||||||||||||
query:    GAAAATCTGATGGCACCCTACAGGAACATGACGGTATCTGCGAGATCCATGTTGCTAAAT

template: ATGCTGAAATATTCGGATTGACCT
          ||||||||||||||||||||||||
query:    ATGCTGAAATATTCGGATTGACCT

# FIB_20
template: ATTCAGACATCAAAAAACTGTTCGGCGAGGTGGATAAGTCGTCCGGTGAGCTGGTGACAC
                                       |||||||||||||||||||||||||||||||
query:    -----------------------------GTGGATAAGTCGTCCGGTGAGCTGGTGACAC

template: TGACACCAAACAATAACAACACCGTACAACCTGTGGCGCTGATGCGTCTGGGCGTCTTTG
          ||||||||||||||||||||||||||||||||||||||||||||||||||||||||||||
query:    TGACACCAAACAATAACAACACCGTACAACCTGTGGCGCTGATGCGTCTGGGCGTCTTTG

template: TACCGACCCTTAAGTCACTGAAGAACAGTAAAAAAAATACACTGTCACGCACCGATGCCA
          ||||||||||||||||||||||||||||||||||||||||||||||||||||||||||||
query:    TACCGACCCTTAAGTCACTGAAGAACAGTAAAAAAAATACACTGTCACGCACCGATGCCA

template: CGGAAGAACTGACGCGTCTTTCTCTGGCCCGTGCAGAAGGATTCGATAAGGTTGAGATCA
          ||||||||||||||||||||||||||||||||||||||||||||||||||||||||||||
query:    CGGAAGAACTGACGCGTCTTTCTCTGGCCCGTGCAGAAGGATTCGATAAGGTTGAGATCA

template: CCGGCCCCCGGCTGGATATGGATAACGATTTCAAGACCTGGGTGGGGATCATTCATTCCT
          ||||||||||||||||||||||||||||||||||||||||||||||||||||||||||||
query:    CCGGCCCCCGGCTGGATATGGATAACGATTTCAAGACCTGGGTGGGGATCATTCATTCCT

template: TTGCCCGCCATAACGTGATTGGTGACAAAGTTGAACTGCCTTTTGTTGAGTTTGCAAAAC
          ||||||||||||||||||||||||||||||||||||||||||||||||||||||||||||
query:    TTGCCCGCCATAACGTGATTGGTGACAAAGTTGAACTGCCTTTTGTTGAGTTTGCAAAAC

template: TGTGTGGTATACC
          |||||||||||||
query:    TGTGTGGTATACC

# FII_1
template: CAAAAACCCCGATAATCTTCACCAGGTTTGGCGACTAAGAGAAGATTACCGGGGCTAACA
          ||||||||||||||||||||||||||||||||||||||||||||||||||||||||||||
query:    CAAAAACCCCGATAATCTTCACCAGGTTTGGCGACTAAGAGAAGATTACCGGGGCTAACA

template: AGAAACTGCATAGAAGCTGTTGCTCTATGCGGGGAGTATAGTTATATGACCGGAAAAGTT
          ||||||||||||||||||||||||||||||||||||||||||||||||||||||||||||
query:    AGAAACTGCATAGAAGCTGTTGCTCTATGCGGGGAGTATAGTTATATGACCGGAAAAGTT

template: CAAGACTTCTTTCTGTGCTCACTCCTTCTGTGCAACA
          |||||||||||||||||||||||||||||||||||||
query:    CAAGACTTCTTTCTGTGCTCACTCCTTCTGTGCAACA
```

Conda version

Long-read sequence

(OV_CH_97_F2_AB-_minion.fasta)

```
pMLST profile: IncF RST

Sequence Type: [F2:A-:B-]
**********************************************************************
Locus     Identity    Coverage    Alignment Length    Allele Length
Gaps    Allele
**********************************************************************
FIA       -           -           -                   -
-       No hit found
FIB       -           -           -                   -
-       No hit found
FIC       -           -           -                   -
-       No hit found
FII       100.0       100.0       157                 157
0       FII_2
FIIK      -           -           -                   -
-       No hit found
FIIS      -           -           -                   -
-       No hit found
FIIY      -           -           -                   -
-       No hit found
======================================================================


Extended Output:

# FII_2
template: CAAAAACCCCGATAATCTTCTTCAACTTTGGCGAGTACGAAAAGATTACCGGGGCCCACT
          ||||||||||||||||||||||||||||||||||||||||||||||||||||||||||||
query:    CAAAAACCCCGATAATCTTCTTCAACTTTGGCGAGTACGAAAAGATTACCGGGGCCCACT

template: TAAACCGTATAGCCAACAATTCAGCTATGCGGGGAGTATAGTTATATGCCCGGAAAAGTT
          ||||||||||||||||||||||||||||||||||||||||||||||||||||||||||||
query:    TAAACCGTATAGCCAACAATTCAGCTATGCGGGGAGTATAGTTATATGCCCGGAAAAGTT

template: CAAGACTTCTTTCTGTGCTCGCTCCTTCTGCGCATTG
          |||||||||||||||||||||||||||||||||||||
query:    CAAGACTTCTTTCTGTGCTCGCTCCTTCTGCGCATTG
```

Docker version

Short-read sequence

(OV_CH_97_Illumina.fasta)

```
pMLST profile: IncF RST

Sequence Type: [F1:A2:B20]
**********************************************************************
Locus    Identity    Coverage    Alignment Length    Allele Length
Gaps    Allele
**********************************************************************
FIA      100.0       100.0       384                 384
0        FIA_2
FIB      100.0       100.0       373                 373
0        FIB_20
FIC      -           -           -                   -
-        No hit found
FII      100.0       100.0       157                 157
0        FII_1!
FII      100.0       100.0       157                 157
0        FII_2!
FIIK     -           -           -                   -
-        No hit found
FIIS     -           -           -                   -
-        No hit found
FIIY     -           -           -                   -
-        No hit found
======================================================================

Notes: ! alleles with multiple perfect hits found, multiple STs might
be found
! FII: Multiple perfect hits found


Extended Output:

# FIA_2
template: TCACCCTCAAATGACAGTCCTGTCTGTGACAAATTGCCCTTAACCCTGTGACAAATTGCC
          ||||||||||||||||||||||||||||||||||||||||||||||||||||||||||||
query:    TCACCCTCAAATGACAGTCCTGTCTGTGACAAATTGCCCTTAACCCTGTGACAAATTGCC

template: CTCAGAAGAAGCTGTTTTTTCACAAAGTTATCCCTGCTTATTGACTCTTTTTTATTTAGT
          ||||||||||||||||||||||||||||||||||||||||||||||||||||||||||||
query:    CTCAGAAGAAGCTGTTTTTTCACAAAGTTATCCCTGCTTATTGACTCTTTTTTATTTAGT

template: GTGACAATCTAAAAACTTGTCACACTTCACATGGATCTGTCATGGCGGAAACAGCGGTTA
          ||||||||||||||||||||||||||||||||||||||||||||||||||||||||||||
query:    GTGACAATCTAAAAACTTGTCACACTTCACATGGATCTGTCATGGCGGAAACAGCGGTTA

template: TCAATCACAAGAAACGTAAAAATAGCCCGCGAATCGTCCAGTCAAACGACCTCACTGAGG
          ||||||||||||||||||||||||||||||||||||||||||||||||||||||||||||
query:    TCAATCACAAGAAACGTAAAAATAGCCCGCGAATCGTCCAGTCAAACGACCTCACTGAGG

template: CGGCATATAGTCTCTCCCGGGATCAAAAACGTATGCTGTATCTGTTCGTTGACCAGATCA
          ||||||||||||||||||||||||||||||||||||||||||||||||||||||||||||
query:    CGGCATATAGTCTCTCCCGGGATCAAAAACGTATGCTGTATCTGTTCGTTGACCAGATCA

template: GAAAATCTGATGGCACCCTACAGGAACATGACGGTATCTGCGAGATCCATGTTGCTAAAT
          ||||||||||||||||||||||||||||||||||||||||||||||||||||||||||||
query:    GAAAATCTGATGGCACCCTACAGGAACATGACGGTATCTGCGAGATCCATGTTGCTAAAT

template: ATGCTGAAATATTCGGATTGACCT
          ||||||||||||||||||||||||
query:    ATGCTGAAATATTCGGATTGACCT

# FIB_20
template: ATTCAGACATCAAAAAACTGTTCGGCGAGGTGGATAAGTCGTCCGGTGAGCTGGTGACAC
          ||||||||||||||||||||||||||||||||||||||||||||||||||||||||||||
query:    ATTCAGACATCAAAAAACTGTTCGGCGAGGTGGATAAGTCGTCCGGTGAGCTGGTGACAC

template: TGACACCAAACAATAACAACACCGTACAACCTGTGGCGCTGATGCGTCTGGGCGTCTTTG
          ||||||||||||||||||||||||||||||||||||||||||||||||||||||||||||
query:    TGACACCAAACAATAACAACACCGTACAACCTGTGGCGCTGATGCGTCTGGGCGTCTTTG

template: TACCGACCCTTAAGTCACTGAAGAACAGTAAAAAAAATACACTGTCACGCACCGATGCCA
          ||||||||||||||||||||||||||||||||||||||||||||||||||||||||||||
query:    TACCGACCCTTAAGTCACTGAAGAACAGTAAAAAAAATACACTGTCACGCACCGATGCCA

template: CGGAAGAACTGACGCGTCTTTCTCTGGCCCGTGCAGAAGGATTCGATAAGGTTGAGATCA
          ||||||||||||||||||||||||||||||||||||||||||||||||||||||||||||
query:    CGGAAGAACTGACGCGTCTTTCTCTGGCCCGTGCAGAAGGATTCGATAAGGTTGAGATCA

template: CCGGCCCCCGGCTGGATATGGATAACGATTTCAAGACCTGGGTGGGGATCATTCATTCCT
          ||||||||||||||||||||||||||||||||||||||||||||||||||||||||||||
query:    CCGGCCCCCGGCTGGATATGGATAACGATTTCAAGACCTGGGTGGGGATCATTCATTCCT

template: TTGCCCGCCATAACGTGATTGGTGACAAAGTTGAACTGCCTTTTGTTGAGTTTGCAAAAC
          ||||||||||||||||||||||||||||||||||||||||||||||||||||||||||||
query:    TTGCCCGCCATAACGTGATTGGTGACAAAGTTGAACTGCCTTTTGTTGAGTTTGCAAAAC

template: TGTGTGGTATACC
          |||||||||||||
query:    TGTGTGGTATACC

# FII_1
template: CAAAAACCCCGATAATCTTCACCAGGTTTGGCGACTAAGAGAAGATTACCGGGGCTAACA
          ||||||||||||||||||||||||||||||||||||||||||||||||||||||||||||
query:    CAAAAACCCCGATAATCTTCACCAGGTTTGGCGACTAAGAGAAGATTACCGGGGCTAACA

template: AGAAACTGCATAGAAGCTGTTGCTCTATGCGGGGAGTATAGTTATATGACCGGAAAAGTT
          ||||||||||||||||||||||||||||||||||||||||||||||||||||||||||||
query:    AGAAACTGCATAGAAGCTGTTGCTCTATGCGGGGAGTATAGTTATATGACCGGAAAAGTT

template: CAAGACTTCTTTCTGTGCTCACTCCTTCTGTGCAACA
          |||||||||||||||||||||||||||||||||||||
query:    CAAGACTTCTTTCTGTGCTCACTCCTTCTGTGCAACA

# FII_2
template: CAAAAACCCCGATAATCTTCTTCAACTTTGGCGAGTACGAAAAGATTACCGGGGCCCACT
          ||||||||||||||||||||||||||||||||||||||||||||||||||||||||||||
query:    CAAAAACCCCGATAATCTTCTTCAACTTTGGCGAGTACGAAAAGATTACCGGGGCCCACT

template: TAAACCGTATAGCCAACAATTCAGCTATGCGGGGAGTATAGTTATATGCCCGGAAAAGTT
          ||||||||||||||||||||||||||||||||||||||||||||||||||||||||||||
query:    TAAACCGTATAGCCAACAATTCAGCTATGCGGGGAGTATAGTTATATGCCCGGAAAAGTT

template: CAAGACTTCTTTCTGTGCTCGCTCCTTCTGCGCATTG
          |||||||||||||||||||||||||||||||||||||
query:    CAAGACTTCTTTCTGTGCTCGCTCCTTCTGCGCATTG
```

Docker version

Long-read sequence

(OV_CH_97_F1_A2_B20_minion.fasta)

```
pMLST profile: IncF RST

Sequence Type: [F1:A2:B20]
**********************************************************************
Locus    Identity    Coverage            Alignment Length    Allele
Length    Gaps    Allele
**********************************************************************
FIA      100.0       100.0               384                 384
0       FIA_2
FIB      100.0       92.22520107238606   344                 373
0       FIB_20?
FIC      -           -                   -                   -
-       No hit found
FII      100.0       100.0               157                 157
0       FII_1
FIIK     -           -                   -                   -
-       No hit found
FIIS     -           -                   -                   -
-       No hit found
FIIY     -           -                   -                   -
-       No hit found
======================================================================

Notes: ? alleles with less than 100% coverage found
? FIB: Uncertain hit, ST can not be trusted.


Extended Output:

# FII_1
template: CAAAAACCCCGATAATCTTCACCAGGTTTGGCGACTAAGAGAAGATTACCGGGGCTAACA
          ||||||||||||||||||||||||||||||||||||||||||||||||||||||||||||
query:    CAAAAACCCCGATAATCTTCACCAGGTTTGGCGACTAAGAGAAGATTACCGGGGCTAACA

template: AGAAACTGCATAGAAGCTGTTGCTCTATGCGGGGAGTATAGTTATATGACCGGAAAAGTT
          ||||||||||||||||||||||||||||||||||||||||||||||||||||||||||||
query:    AGAAACTGCATAGAAGCTGTTGCTCTATGCGGGGAGTATAGTTATATGACCGGAAAAGTT

template: CAAGACTTCTTTCTGTGCTCACTCCTTCTGTGCAACA
          |||||||||||||||||||||||||||||||||||||
query:    CAAGACTTCTTTCTGTGCTCACTCCTTCTGTGCAACA

# FIB_20
template: ATTCAGACATCAAAAAACTGTTCGGCGAGGTGGATAAGTCGTCCGGTGAGCTGGTGACAC
                                       |||||||||||||||||||||||||||||||
query:    -----------------------------GTGGATAAGTCGTCCGGTGAGCTGGTGACAC

template: TGACACCAAACAATAACAACACCGTACAACCTGTGGCGCTGATGCGTCTGGGCGTCTTTG
          ||||||||||||||||||||||||||||||||||||||||||||||||||||||||||||
query:    TGACACCAAACAATAACAACACCGTACAACCTGTGGCGCTGATGCGTCTGGGCGTCTTTG

template: TACCGACCCTTAAGTCACTGAAGAACAGTAAAAAAAATACACTGTCACGCACCGATGCCA
          ||||||||||||||||||||||||||||||||||||||||||||||||||||||||||||
query:    TACCGACCCTTAAGTCACTGAAGAACAGTAAAAAAAATACACTGTCACGCACCGATGCCA

template: CGGAAGAACTGACGCGTCTTTCTCTGGCCCGTGCAGAAGGATTCGATAAGGTTGAGATCA
          ||||||||||||||||||||||||||||||||||||||||||||||||||||||||||||
query:    CGGAAGAACTGACGCGTCTTTCTCTGGCCCGTGCAGAAGGATTCGATAAGGTTGAGATCA

template: CCGGCCCCCGGCTGGATATGGATAACGATTTCAAGACCTGGGTGGGGATCATTCATTCCT
          ||||||||||||||||||||||||||||||||||||||||||||||||||||||||||||
query:    CCGGCCCCCGGCTGGATATGGATAACGATTTCAAGACCTGGGTGGGGATCATTCATTCCT

template: TTGCCCGCCATAACGTGATTGGTGACAAAGTTGAACTGCCTTTTGTTGAGTTTGCAAAAC
          ||||||||||||||||||||||||||||||||||||||||||||||||||||||||||||
query:    TTGCCCGCCATAACGTGATTGGTGACAAAGTTGAACTGCCTTTTGTTGAGTTTGCAAAAC

template: TGTGTGGTATACC
          |||||||||||||
query:    TGTGTGGTATACC

# FIA_2
template: TCACCCTCAAATGACAGTCCTGTCTGTGACAAATTGCCCTTAACCCTGTGACAAATTGCC
          ||||||||||||||||||||||||||||||||||||||||||||||||||||||||||||
query:    TCACCCTCAAATGACAGTCCTGTCTGTGACAAATTGCCCTTAACCCTGTGACAAATTGCC

template: CTCAGAAGAAGCTGTTTTTTCACAAAGTTATCCCTGCTTATTGACTCTTTTTTATTTAGT
          ||||||||||||||||||||||||||||||||||||||||||||||||||||||||||||
query:    CTCAGAAGAAGCTGTTTTTTCACAAAGTTATCCCTGCTTATTGACTCTTTTTTATTTAGT

template: GTGACAATCTAAAAACTTGTCACACTTCACATGGATCTGTCATGGCGGAAACAGCGGTTA
          ||||||||||||||||||||||||||||||||||||||||||||||||||||||||||||
query:    GTGACAATCTAAAAACTTGTCACACTTCACATGGATCTGTCATGGCGGAAACAGCGGTTA

template: TCAATCACAAGAAACGTAAAAATAGCCCGCGAATCGTCCAGTCAAACGACCTCACTGAGG
          ||||||||||||||||||||||||||||||||||||||||||||||||||||||||||||
query:    TCAATCACAAGAAACGTAAAAATAGCCCGCGAATCGTCCAGTCAAACGACCTCACTGAGG

template: CGGCATATAGTCTCTCCCGGGATCAAAAACGTATGCTGTATCTGTTCGTTGACCAGATCA
          ||||||||||||||||||||||||||||||||||||||||||||||||||||||||||||
query:    CGGCATATAGTCTCTCCCGGGATCAAAAACGTATGCTGTATCTGTTCGTTGACCAGATCA

template: GAAAATCTGATGGCACCCTACAGGAACATGACGGTATCTGCGAGATCCATGTTGCTAAAT
          ||||||||||||||||||||||||||||||||||||||||||||||||||||||||||||
query:    GAAAATCTGATGGCACCCTACAGGAACATGACGGTATCTGCGAGATCCATGTTGCTAAAT

template: ATGCTGAAATATTCGGATTGACCT
          ||||||||||||||||||||||||
query:    ATGCTGAAATATTCGGATTGACCT
```

Docker version

Long-read sequence

(OV_CH_97_F2_AB-_minion.fasta)

```
pMLST profile: IncF RST

Sequence Type: [F2:A-:B-]
**********************************************************************
Locus    Identity    Coverage    Alignment Length    Allele Length
Gaps    Allele
**********************************************************************
FIA      -           -           -                   -
-       No hit found
FIB      -           -           -                   -
-       No hit found
FIC      -           -           -                   -
-       No hit found
FII      100.0       100.0       157                 157
0       FII_2
FIIK     -           -           -                   -
-       No hit found
FIIS     -           -           -                   -
-       No hit found
FIIY     -           -           -                   -
-       No hit found
======================================================================


Extended Output:

# FII_2
template: CAAAAACCCCGATAATCTTCTTCAACTTTGGCGAGTACGAAAAGATTACCGGGGCCCACT
          ||||||||||||||||||||||||||||||||||||||||||||||||||||||||||||
query:    CAAAAACCCCGATAATCTTCTTCAACTTTGGCGAGTACGAAAAGATTACCGGGGCCCACT

template: TAAACCGTATAGCCAACAATTCAGCTATGCGGGGAGTATAGTTATATGCCCGGAAAAGTT
          ||||||||||||||||||||||||||||||||||||||||||||||||||||||||||||
query:    TAAACCGTATAGCCAACAATTCAGCTATGCGGGGAGTATAGTTATATGCCCGGAAAAGTT

template: CAAGACTTCTTTCTGTGCTCGCTCCTTCTGCGCATTG
          |||||||||||||||||||||||||||||||||||||
query:    CAAGACTTCTTTCTGTGCTCGCTCCTTCTGCGCATTG
```

<u>CGE web version</u>

Short-read sequence

(OV_CH_97_Illumina.fasta)

pMLST profile: *IncF RST*

Sequence Type: *[F2:A2:B20]*

Locus	Identity	Coverage	Alignment Length	Allele Length	Gaps	Allele
FIA	100.0	100.0	384	384	0	FIA_2
FIB	100.0	100.0	373	373	0	FIB_20
FIC	97.35099337748345	75.0	151	200	1	FIC_3?*
FII	100.0	100.0	157	157	0	FII_2!
FII	100.0	100.0	157	157	0	FII_1!
FIIK						No hit found
FIIS						No hit found
FIIY						No hit found

Notes: ?* alleles with less than 100% identity and 100% coverages found

! FII: *Multiple perfect hits found*

?* FIC: *Imperfect hit, ST can not be trusted!*

CGE web version

Long-read sequence

(OV_CH_97_F1_A2_B20_minion.fasta)

pMLST profile: *IncF RST*

Sequence Type: *[F1:A2:B20]*

Locus	Identity	Coverage	Alignment Length	Allele Length	Gaps	Allele
FIA	100.0	100.0	384	384	0	FIA_2
FIB	100.0	92.22520107238606	344	373	0	FIB_20?
FIC	97.35099337748345	75.0	151	200	1	FIC_3?*
FII	100.0	100.0	157	157	0	FII_1
FIIK						No hit found
FIIS						No hit found
FIIY						No hit found

Notes: ?* alleles with less than 100% identity and 100% coverages found

? FIB: *Uncertain hit, ST can not be trusted.*

?* FIC: *Imperfect hit, ST can not be trusted!*

CGE web version

Long-read sequence

(OV_CH_97_F2_AB-_minion.fasta)

pMLST profile: *IncF RST*

Sequence Type: *[F2:A-:B-]*

Locus	Identity	Coverage	Alignment Length	Allele Length	Gaps	Allele
FIA						No hit found
FIB						No hit found
FIC	95.1048951048951	71.0	143	200	1	FIC_5?*
FII	100.0	100.0	157	157	0	FII_2
FIIK						No hit found
FIIS						No hit found
FIIY						No hit found

Notes: ?* alleles with less than 100% identity and 100% coverages found

?* FIC: *Imperfect hit, ST can not be trusted!*
