# Supplementary material for: The presence of multiple variants of IncF plasmid alleles in a single genome sequence can hinder accurate replicon sequence typing using in silico pMLST tools
Source: mSystems. 2025 Apr 8;10(5):e01010-24. doi: 10.1128/msystems.01010-24 (PMC12090814; doi:10.1128/msystems.01010-24)
Supplement: Data S3 — Full output of three pMLST tool variants displaying the C4/F18 and B1/B58 situations (Fig. 3 and 4). [file msystems.01010-24-s0003.pdf]

Supplement data S3. Full output of three pMLST tool variants displaying the C4/F18 and B1/B58 situations (Fig. 3, Fig. 4)

Conda version

Short-read sequence

(B0009170_Illumina.fasta)

```
pMLST profile: IncF RST

Sequence Type: [C4:A-:B1]
*******************************************************************************************
Locus    Identity    Coverage    Alignment Length    Allele Length    Gaps    Allele
*******************************************************************************************
FIA      -           -           -                   -                -       No hit found
FIB      100.0       100.0       373                 373              0       FIB_1
FIC      100.0       100.0       200                 200              0       FIC_4
FII      -           -           -                   -                -       No hit found
FIIK     -           -           -                   -                -       No hit found
FIIS     -           -           -                   -                -       No hit found
FIIY     -           -           -                   -                -       No hit found
===========================================================================================


Extended Output:

# FIB_1
template: ATTCAGACATCAAAAAACTGTTCGGCGAGGTGGATAAGTCCTCCGGTGAGCTGGTGACAC
          ||||||||||||||||||||||||||||||||||||||||||||||||||||||||||||
query:    ATTCAGACATCAAAAAACTGTTCGGCGAGGTGGATAAGTCCTCCGGTGAGCTGGTGACAC

template: TGACACCAAACAATAACAACACCGTACAACCTGTGGCGCTGATGCGTCTGGGCGTTTTTG
          ||||||||||||||||||||||||||||||||||||||||||||||||||||||||||||
query:    TGACACCAAACAATAACAACACCGTACAACCTGTGGCGCTGATGCGTCTGGGCGTTTTTG

template: TACCGACCCTTAAATCACTGAAGAACAGTAAAAAAAATACACTGTCACGTACTGATGCCA
          ||||||||||||||||||||||||||||||||||||||||||||||||||||||||||||
query:    TACCGACCCTTAAATCACTGAAGAACAGTAAAAAAAATACACTGTCACGTACTGATGCCA

template: CGGAAGAGCTGACACGTCTTTCCCTGGCCCGTGCTGAGGGATTCGATAAGGTTGAGATCA
          ||||||||||||||||||||||||||||||||||||||||||||||||||||||||||||
query:    CGGAAGAGCTGACACGTCTTTCCCTGGCCCGTGCTGAGGGATTCGATAAGGTTGAGATCA

template: CCGGCCCCCGCCTGGATATGGATAATGATTTCAAGACCTGGGTGGGGATCATTCATTCCT
          ||||||||||||||||||||||||||||||||||||||||||||||||||||||||||||
query:    CCGGCCCCCGCCTGGATATGGATAATGATTTCAAGACCTGGGTGGGGATCATTCATTCCT

template: TTGCCCGCCATAACGTGATTGGTGACAAAGTTGAACTGCCTTTTGTTGAGTTTGCAAAAC
          ||||||||||||||||||||||||||||||||||||||||||||||||||||||||||||
query:    TTGCCCGCCATAACGTGATTGGTGACAAAGTTGAACTGCCTTTTGTTGAGTTTGCAAAAC

template: TGTGTGGTATACC
          |||||||||||||
query:    TGTGTGGTATACC

# FIC_4
template: CAGAGCTGAAACGCAGAGCGACTTCGTAAATATTCACATTCTTGCTTATCTCAGGAGTGA
          ||||||||||||||||||||||||||||||||||||||||||||||||||||||||||||
query:    CAGAGCTGAAACGCAGAGCGACTTCGTAAATATTCACATTCTTGCTTATCTCAGGAGTGA

template: GTGGTAGATTGCTGATCGTTTAAGGAATTTTGTGGCTGGCCACGCCGTAAGGTGGCAGGG
          ||||||||||||||||||||||||||||||||||||||||||||||||||||||||||||
query:    GTGGTAGATTGCTGATCGTTTAAGGAATTTTGTGGCTGGCCACGCCGTAAGGTGGCAGGG

template: AACTGGTTCTGATGTGGATTTACAGGAGCCAGAAAAGCGAAAACCCCGATAATCTTCTTC
          ||||||||||||||||||||||||||||||||||||||||||||||||||||||||||||
query:    AACTGGTTCTGATGTGGATTTACAGGAGCCAGAAAAGCGAAAACCCCGATAATCTTCTTC

template: AAGTTTGGCGACTAGAAAGA
          ||||||||||||||||||||
query:    AAGTTTGGCGACTAGAAAGA
```

<u>Conda version</u>

Long-read sequence

(B0009170_F18-C4_A_B1_minion.fasta)

```
pMLST profile: IncF RST

Sequence Type: [C4:A-:B58]
**************************************************************************************************
Locus    Identity    Coverage           Alignment Length    Allele Length    Gaps    Allele
**************************************************************************************************
FIA      -           -                  -                   -                -       No hit found
FIB      100.0       92.22520107238606  344                 373              0       FIB_58?
FIC      100.0       100.0              200                 200              0       FIC_4
FII      -           -                  -                   -                -       No hit found
FIIK     -           -                  -                   -                -       No hit found
FIIS     -           -                  -                   -                -       No hit found
FIIY     -           -                  -                   -                -       No hit found
==================================================================================================

Notes: ? alleles with less than 100% coverage found
? FIB: Uncertain hit, ST can not be trusted.


Extended Output:

# FIB_58
template: ATTCAGACATAAAAAAACTGTTCGGCGAGGTGGATAAGTCCTCCGGTGAGCTGGTGACAC
                                       |||||||||||||||||||||||||||||||
query:    -----------------------------GTGGATAAGTCCTCCGGTGAGCTGGTGACAC

template: TGACACCAAACAATAACAACACCGTACAACCTGTGGCGCTGATGCGTCTGGGCGTTTTTG
          ||||||||||||||||||||||||||||||||||||||||||||||||||||||||||||
query:    TGACACCAAACAATAACAACACCGTACAACCTGTGGCGCTGATGCGTCTGGGCGTTTTTG

template: TACCGACCCTTAAATCACTGAAGAACAGTAAAAAAAATACACTGTCACGTACTGATGCCA
          ||||||||||||||||||||||||||||||||||||||||||||||||||||||||||||
query:    TACCGACCCTTAAATCACTGAAGAACAGTAAAAAAAATACACTGTCACGTACTGATGCCA

template: CGGAAGAGCTGACACGTCTTTCCCTGGCCCGTGCTGAGGGATTCGATAAGGTTGAGATCA
          ||||||||||||||||||||||||||||||||||||||||||||||||||||||||||||
query:    CGGAAGAGCTGACACGTCTTTCCCTGGCCCGTGCTGAGGGATTCGATAAGGTTGAGATCA

template: CCGGCCCCCGCCTGGATATGGATAATGATTTCAAGACCTGGGTGGGGATCATTCATTCCT
          ||||||||||||||||||||||||||||||||||||||||||||||||||||||||||||
query:    CCGGCCCCCGCCTGGATATGGATAATGATTTCAAGACCTGGGTGGGGATCATTCATTCCT

template: TTGCCCGCCATAACGTGATTGGTGACAAAGTTGAACTGCCTTTTGTTGAGTTTGCAAAAC
          ||||||||||||||||||||||||||||||||||||||||||||||||||||||||||||
query:    TTGCCCGCCATAACGTGATTGGTGACAAAGTTGAACTGCCTTTTGTTGAGTTTGCAAAAC

template: TGTGTGGTATACC
          |||||||||||||
query:    TGTGTGGTATACC

# FIC_4
template: CAGAGCTGAAACGCAGAGCGACTTCGTAAATATTCACATTCTTGCTTATCTCAGGAGTGA
          ||||||||||||||||||||||||||||||||||||||||||||||||||||||||||||
query:    CAGAGCTGAAACGCAGAGCGACTTCGTAAATATTCACATTCTTGCTTATCTCAGGAGTGA

template: GTGGTAGATTGCTGATCGTTTAAGGAATTTTGTGGCTGGCCACGCCGTAAGGTGGCAGGG
          ||||||||||||||||||||||||||||||||||||||||||||||||||||||||||||
query:    GTGGTAGATTGCTGATCGTTTAAGGAATTTTGTGGCTGGCCACGCCGTAAGGTGGCAGGG

template: AACTGGTTCTGATGTGGATTTACAGGAGCCAGAAAAGCGAAAACCCCGATAATCTTCTTC
          ||||||||||||||||||||||||||||||||||||||||||||||||||||||||||||
query:    AACTGGTTCTGATGTGGATTTACAGGAGCCAGAAAAGCGAAAACCCCGATAATCTTCTTC

template: AAGTTTGGCGACTAGAAAGA
          ||||||||||||||||||||
query:    AAGTTTGGCGACTAGAAAGA
```

Docker version

Short-read sequence

(B0009170_Illumina.fasta)

```
pMLST profile: IncF RST

Sequence Type: [C4:A-:B1]
********************************************************************************************
Locus     Identity    Coverage    Alignment Length    Allele Length    Gaps    Allele
********************************************************************************************
FIA       -           -           -                   -                -       No hit found
FIB       100.0       100.0       373                 373              0       FIB_1
FIC       100.0       100.0       200                 200              0       FIC_4
FII       -           -           -                   -                -       No hit found
FIIK      -           -           -                   -                -       No hit found
FIIS      -           -           -                   -                -       No hit found
FIIY      -           -           -                   -                -       No hit found
============================================================================================

Extended Output:

# FIB_1
template: ATTCAGACATCAAAAAACTGTTCGGCGAGGTGGATAAGTCCTCCGGTGAGCTGGTGACAC
          ||||||||||||||||||||||||||||||||||||||||||||||||||||||||||||
query:    ATTCAGACATCAAAAAACTGTTCGGCGAGGTGGATAAGTCCTCCGGTGAGCTGGTGACAC

template: TGACACCAAACAATAACAACACCGTACAACCTGTGGCGCTGATGCGTCTGGGCGTTTTTG
          ||||||||||||||||||||||||||||||||||||||||||||||||||||||||||||
query:    TGACACCAAACAATAACAACACCGTACAACCTGTGGCGCTGATGCGTCTGGGCGTTTTTG

template: TACCGACCCTTAAATCACTGAAGAACAGTAAAAAAAAATACACTGTCACGTACTGATGCCA
          ||||||||||||||||||||||||||||||||||||||||||||||||||||||||||||
query:    TACCGACCCTTAAATCACTGAAGAACAGTAAAAAAAAATACACTGTCACGTACTGATGCCA

template: CGGAAGAGCTGACACGTCTTTCCCTGGCCCGTGCTGAGGGATTCGATAAGGTTGAGATCA
          ||||||||||||||||||||||||||||||||||||||||||||||||||||||||||||
query:    CGGAAGAGCTGACACGTCTTTCCCTGGCCCGTGCTGAGGGATTCGATAAGGTTGAGATCA

template: CCGGCCCCCGCCTGGATATGGATAATGATTTCAAGACCTGGGTGGGGATCATTCATTCCT
          ||||||||||||||||||||||||||||||||||||||||||||||||||||||||||||
query:    CCGGCCCCCGCCTGGATATGGATAATGATTTCAAGACCTGGGTGGGGATCATTCATTCCT

template: TTGCCCGCCATAACGTGATTGGTGACAAAGTTGAACTGCCTTTTGTTGAGTTTGCAAAAC
          ||||||||||||||||||||||||||||||||||||||||||||||||||||||||||||
query:    TTGCCCGCCATAACGTGATTGGTGACAAAGTTGAACTGCCTTTTGTTGAGTTTGCAAAAC

template: TGTGTGGTATACC
          |||||||||||||
query:    TGTGTGGTATACC

# FIC_4
template: CAGAGCTGAAACGCAGAGCGACTTCGTAAATATTCACATTCTTGCTTATCTCAGGAGTGA
          ||||||||||||||||||||||||||||||||||||||||||||||||||||||||||||
query:    CAGAGCTGAAACGCAGAGCGACTTCGTAAATATTCACATTCTTGCTTATCTCAGGAGTGA

template: GTGGTAGATTGCTGATCGTTTAAGGAATTTTGTGGCTGGCCACGCCGTAAGGTGGCAGGG
          ||||||||||||||||||||||||||||||||||||||||||||||||||||||||||||
query:    GTGGTAGATTGCTGATCGTTTAAGGAATTTTGTGGCTGGCCACGCCGTAAGGTGGCAGGG

template: AACTGGTTCTGATGTGGATTTACAGGAGCCAGAAAAGCGAAAACCCCGATAATCTTCTTC
          ||||||||||||||||||||||||||||||||||||||||||||||||||||||||||||
query:    AACTGGTTCTGATGTGGATTTACAGGAGCCAGAAAAGCGAAAACCCCGATAATCTTCTTC

template: AAGTTTGGCGACTAGAAAGA
          ||||||||||||||||||||
query:    AAGTTTGGCGACTAGAAAGA
```

Docker version

Long-read sequence

(B0009170_F18-C4_A_B1_minion.fasta)

```
pMLST profile: IncF RST

Sequence Type: [C4:A-:B1]
**************************************************************************************************
Locus    Identity    Coverage           Alignment Length    Allele Length    Gaps    Allele
**************************************************************************************************
FIA      -           -                  -                   -                -       No hit found
FIB      100.0       92.22520107238606  344                 373              0       FIB_1?
FIC      100.0       100.0              200                 200              0       FIC_4
FII      -           -                  -                   -                -       No hit found
FIIK     -           -                  -                   -                -       No hit found
FIIS     -           -                  -                   -                -       No hit found
FIIY     -           -                  -                   -                -       No hit found
==================================================================================================

Notes: ? alleles with less than 100% coverage found
? FIB: Uncertain hit, ST can not be trusted.


Extended Output:

# FIB_1
template: ATTCAGACATCAAAAAACTGTTCGGCGAGGTGGATAAGTCCTCCGGTGAGCTGGTGACAC
                                       |||||||||||||||||||||||||||||||
query:    -----------------------------GTGGATAAGTCCTCCGGTGAGCTGGTGACAC

template: TGACACCAAACAATAACAACACCGTACAACCTGTGGCGCTGATGCGTCTGGGCGTTTTTG
          ||||||||||||||||||||||||||||||||||||||||||||||||||||||||||||
query:    TGACACCAAACAATAACAACACCGTACAACCTGTGGCGCTGATGCGTCTGGGCGTTTTTG

template: TACCGACCCTTAAATCACTGAAGAACAGTAAAAAAAATACACTGTCACGTACTGATGCCA
          ||||||||||||||||||||||||||||||||||||||||||||||||||||||||||||
query:    TACCGACCCTTAAATCACTGAAGAACAGTAAAAAAAATACACTGTCACGTACTGATGCCA

template: CGGAAGAGCTGACACGTCTTTCCCTGGCCCGTGCTGAGGGATTCGATAAGGTTGAGATCA
          ||||||||||||||||||||||||||||||||||||||||||||||||||||||||||||
query:    CGGAAGAGCTGACACGTCTTTCCCTGGCCCGTGCTGAGGGATTCGATAAGGTTGAGATCA

template: CCGGCCCCCGCCTGGATATGGATAATGATTTCAAGACCTGGGTGGGGATCATTCATTCCT
          ||||||||||||||||||||||||||||||||||||||||||||||||||||||||||||
query:    CCGGCCCCCGCCTGGATATGGATAATGATTTCAAGACCTGGGTGGGGATCATTCATTCCT

template: TTGCCCGCCATAACGTGATTGGTGACAAAGTTGAACTGCCTTTTGTTGAGTTTGCAAAAC
          ||||||||||||||||||||||||||||||||||||||||||||||||||||||||||||
query:    TTGCCCGCCATAACGTGATTGGTGACAAAGTTGAACTGCCTTTTGTTGAGTTTGCAAAAC

template: TGTGTGGTATACC
          |||||||||||||
query:    TGTGTGGTATACC

# FIC_4
template: CAGAGCTGAAACGCAGAGCGACTTCGTAAATATTCACATTCTTGCTTATCTCAGGAGTGA
          ||||||||||||||||||||||||||||||||||||||||||||||||||||||||||||
query:    CAGAGCTGAAACGCAGAGCGACTTCGTAAATATTCACATTCTTGCTTATCTCAGGAGTGA

template: GTGGTAGATTGCTGATCGTTTAAGGAATTTTGTGGCTGGCCACGCCGTAAGGTGGCAGGG
          ||||||||||||||||||||||||||||||||||||||||||||||||||||||||||||
query:    GTGGTAGATTGCTGATCGTTTAAGGAATTTTGTGGCTGGCCACGCCGTAAGGTGGCAGGG

template: AACTGGTTCTGATGTGGATTTACAGGAGCCAGAAAAGCGAAAACCCCGATAATCTTCTTC
          ||||||||||||||||||||||||||||||||||||||||||||||||||||||||||||
query:    AACTGGTTCTGATGTGGATTTACAGGAGCCAGAAAAGCGAAAACCCCGATAATCTTCTTC

template: AAGTTTGGCGACTAGAAAGA
          ||||||||||||||||||||
query:    AAGTTTGGCGACTAGAAAGA
```

<u>CGE web version</u>

Short-read sequence

(B0009170_Illumina.fasta)

pMLST profile: *IncF RST*

Sequence Type: *[F18:A-:B1]*

Locus	Identity	Coverage	Alignment Length	Allele Length	Gaps	Allele
FIA						No hit found
FIB	100.0	100.0	373	373	0	FIB_1
FIC	100.0	100.0	200	200	0	FIC_4
FII	100.0	100.0	155	155	0	FII_18
FIIK						No hit found
FIIS						No hit found
FIIY						No hit found

CGE web version

Long-read sequence

(B0009170_F18-C4_A_B1_minion.fasta)

1st run

pMLST profile: *IncF RST*

Sequence Type: *[F18:A-:B58]*

Locus	Identity	Coverage	Alignment Length	Allele Length	Gaps	Allele
FIA						No hit found
FIB	100.0	92.22520107238606	344	373	0	FIB_58?
FIC	100.0	100.0	200	200	0	FIC_4
FII	100.0	100.0	155	155	0	FII_18
FIIK						No hit found
FIIS						No hit found
FIIY						No hit found

Notes: ? alleles with less than 100% coverage found

? FIB: *Uncertain hit, ST can not be trusted.*

2nd run

pMLST profile: *IncF RST*

Sequence Type: *[F18:A-:B1]*

Locus	Identity	Coverage	Alignment Length	Allele Length	Gaps	Allele
FIA						No hit found
FIB	100.0	92.22520107238606	344	373	0	FIB_1?
FIC	100.0	100.0	200	200	0	FIC_4
FII	100.0	100.0	155	155	0	FII_18
FIIK						No hit found
FIIS						No hit found
FIIY						No hit found

Notes: ? alleles with less than 100% coverage found

? FIB: *Uncertain hit, ST can not be trusted.*
